# ID1 in hematopoiesis and hematologic disorders: novel potentials of a classic differentiation regulator

**DOI:** 10.1186/s11658-025-00801-y

**Published:** 2025-10-27

**Authors:** Yangjing Zhao, Jiaxin Xu, Yue You, Hui Qian, Jingdong Zhou, Jun Qian

**Affiliations:** 1 Jiangsu Key Laboratory of Medical Science and Laboratory Medicine, School of Medicine, Jiangsu University, Zhenjiang, 212013 China; 2 Institute of Hematology, Jiangsu University, Zhenjiang, 212013 China; 3 Department of Hematology, Affiliated People’s Hospital of Jiangsu University, Zhenjiang, 212002 China

**Keywords:** Inhibitor of DNA binding 1, Hematopoiesis, Differentiation, Hematologic disorders, Antileukemic effect

## Abstract

The basic helix-loop-helix (bHLH) transcription factor family plays a crucial role in regulating cellular differentiation and development. Inhibitor of DNA binding 1 (ID1), which lacks a DNA-binding motif, functions as a dominant-negative inhibitor of class I and II bHLH factors to antagonize their abilities to bind to DNA and transcriptionally regulate target genes. Given that hematopoiesis is a dynamic and intricate process involving the differentiation of hematopoietic stem and progenitor cells into mature lineage cell types, elucidating the regulatory role of ID1 as a differentiation inhibitor within the hematopoietic system is paramount. Physiologically, ID1 is indispensable for maintaining normal bone marrow function and cell fate determination. However, aberrant ID1 expression, driven by pathogenic mechanisms, such as gene mutations or oncogenic kinases, contributes to the initiation and progression of various blood disorders, particularly leukemia. In this review, we comprehensively summarize the expression patterns of ID1 in hematopoietic and stromal cells within the bone marrow niche, and delve into its modulation of blood lineage commitment and development. While some discrepancies in the literature may arise from differences in experimental models or detection methods, it is evident that precise ID1 regulation is crucial for myeloid-lymphoid fate decisions. Moreover, ID1 overexpression is a causal factor in hematologic malignancies. Encouragingly, significant strides have yielded promising antileukemic effects of ID1 inhibitors, both alone and in combination with targeted therapies against oncogenic signaling pathways. Nevertheless, further efforts are needed to develop innovative and practical strategies that modulate ID1 activity to restore and sustain hematopoietic homeostasis.

**Graphical Abstract:**

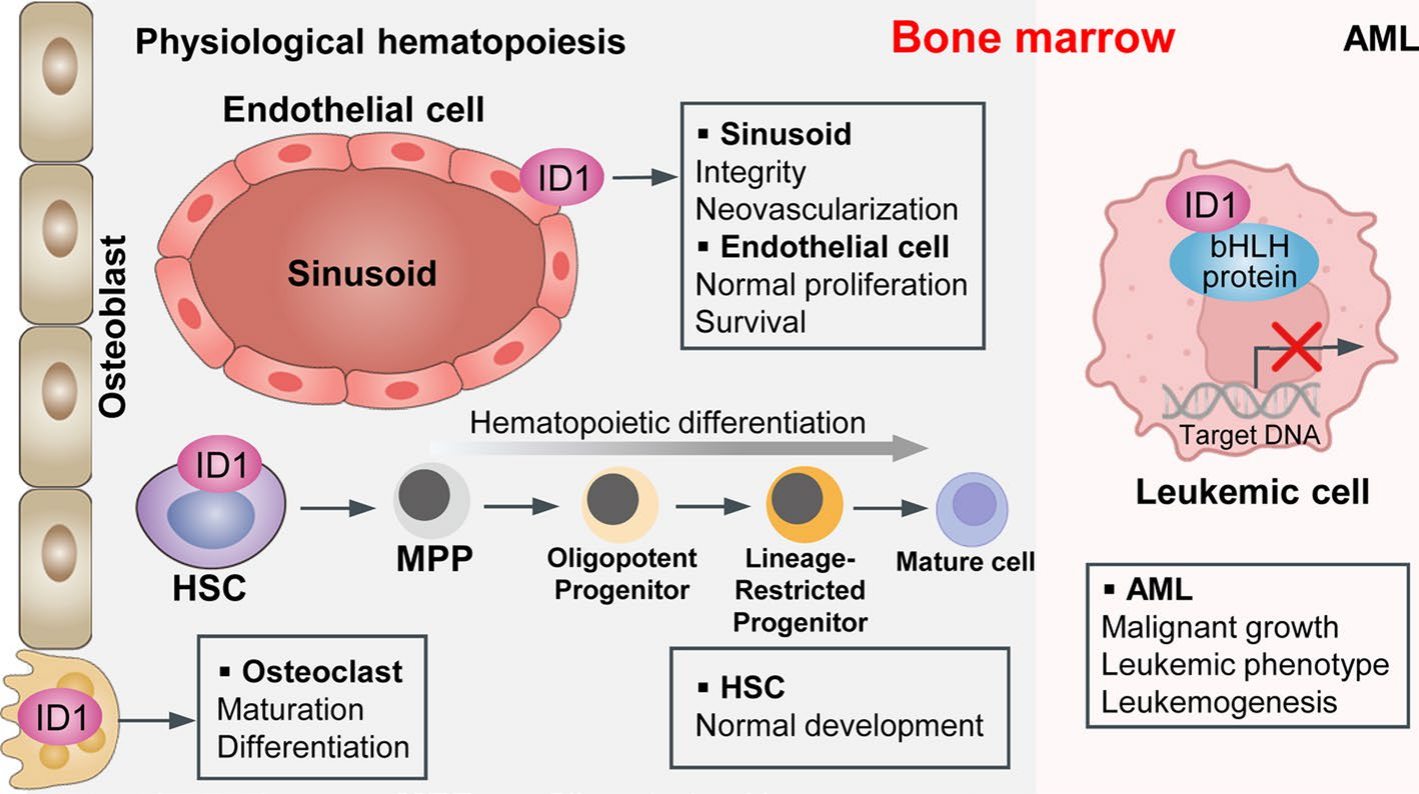

## Introduction

**Fig. 1:**
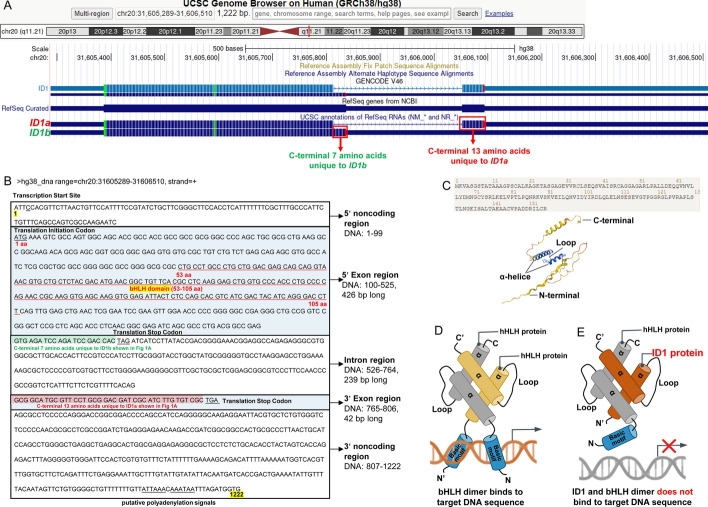
Gene structure and sequence of *ID1*. **A** The chromosomal localization, genomic coordinate, and two transcripts (*ID1a* and *ID1b*) of the human *ID1* gene visualized in the UCSC Genome Browser (https://genome.ucsc.edu/, accessed on 1 October 2024). *ID1a* has unique 13 c-terminal amino acids encoded by the 3′ exon, and *ID1b* has an additional seven c-terminal amino acids. **B** DNA sequence of the human *ID1* gene. *ID1* gene contains 1222 base pairs and two exons, the 5′ exon (426 bp) and 3′ exon (42 bp) separated by an intron of 239 bp. The untranslated sequence is shown with no spaces, and the translated sequence is displayed as triplets. The red underline indicates the HLH domain (100–525 aa) within the first exon. The C-terminal 13 amino acids encoded by the second exon unique to *ID1a* and seven amino acids generated by alternative splicing unique to *ID1b* are highlighted in red and green, respectively. Other essential gene elements are underlined in black and indicated as described below the sequence. **C** The protein sequence and AlphaFold structure of ID1 protein from the AlphaFold database (https://alphafold.ebi.ac.uk/entry/P41134, accessed on 1 October 2024). **D** The N-terminal basic DNA-binding motifs of the homologous or heterodimerized two bHLH proteins bind and generally activate target gene transcription. **E** The dimerization of ID1 and bHLH proteins weakens the DNA-binding ability of the bHLH protein to target DNA sequence

The helix-loop-helix (HLH) is the third-largest transcription factor family in the human genome, comprising more than 200 members that share a highly conserved HLH dimerization domain and a basic DNA-binding region. Thus, HLH protein is also known as basic-HLH (bHLH) protein. On the basis of their distinct dimerization properties, the DNA-binding specificity, and tissue distributions, HLH proteins are classified into seven major categories (I-VII). The HLH domain consists of two conserved amphiphilic α-helices connected by a loop, which mediates the homodimerization or heterodimerization of HLH proteins. Upon dimerization, the N-terminal basic DNA-binding motif folds into a tweezers-like structure, enabling interaction with specific DNA sequences to regulate gene transcription during cellular development and differentiation [1]. These consensus DNA motifs include E-box (CANNTG), N-box (CACNAG), and Ets site (GGAA/T) [2]. Class I bHLH transcription factors are widely expressed E-proteins like transcription factor 3 (TCF3, also named E2A, E12/E47 and ITF1), TCF4 (also named E2-2 and ITF2) and TCF12 (also named HEB). Class II factors are tissue-specific myogenic regulatory factors, e.g., MYOD1. Inhibitor of DNA binding (ID), also called inhibitor of DNA binding and cell differentiation, was initially identified by Benezra et al. in 1990 and belongs to class V HLH factors distinguished by the absence of a DNA-binding motif. ID proteins preferentially interact with other bHLH proteins (primarily class I factors and occasionally class II) to generate inactive heterodimers. As a result, ID proteins attenuate the DNA-binding ability of these proteins, acting as dominant negative HLH transcription factors (Fig. 1, all figures are created with BioRender.com.) [3, 4]. Currently, four members (*ID1-4*) of the ID family have been identified in mammals and are located on human chromosomes 20q11 [5], 2p25 [6], 1p36.1 [7], and 6p21.3–22 [8], respectively, among which *ID1* is the best-characterized member.

The *ID1* gene is located near the centromere at human chromosome 20q11.21 and encompasses a total length of 1222 base pairs (bp). The 426 bp 5′ exon containing the HLH region and the 42 bp 3′ exon are separated by a single 239 bp intron. The *ID1* gene gives rise to two transcripts, denoted as the longer variant (*ID1a*) and truncated form (*ID1b*), yielding mRNA transcripts represented by accession numbers NM_002165.4 (protein NP_002156.2) and NM_181353.3 (protein NP_851998.1), respectively. *ID1b* is generated by alternative splicing, skipping the 5′ splicing donor signal that follows the 5′ exon. This skipping adds a 24 bp translatable sequence to *ID1b*, resulting in an additional 7 c-terminal amino acids, but a lack of 13 c-terminal amino acids encoded by the 3′ exon unique to *ID1a* [5]. Consequently, the resultant protein products of *ID1a* and *ID1b* have 155 and 149 amino acids in length, respectively. Despite this divergence at the C-terminus, the two isoforms exhibit identical characteristics within the 5′ exon housing the HLH domain (amino acid residues 53 through 105) (Fig. 1). These two *ID1* isoforms have also been detected in leukemic cell lysates, but their functional differences still need to be elucidated [9].

Over the past thirty years, researchers have unveiled the multifaceted involvement of ID1 in various biological processes integral to cellular differentiation, proliferation, cell cycle regulation, stem cell identity preservation, and malignancy transformation. ID1 exerts a wide range of functions in regulating the balance of proliferation and differentiation in hematopoietic, neuronal, muscle, and other cell types [10–15]. ID1 has been well recognized as a key physiological regulator required for normal hematopoiesis function in vivo, and its expression is critical to determine hematopoietic cell fate. The hematopoietic microenvironment in the bone marrow (BM), termed as niche, consists of hematopoietic stem cells (HSCs) and HSC-derived cells (e.g., megakaryocytes and macrophages) and nonhematopoietic stromal cells (e.g., endothelial cells, perivascular mesenchymal stem cells (MSCs), osteoclast, osteoblast, osteoprogenitor, adipocytes and sinusoids) [16]. In adult mammals, hematopoiesis occurs in the BM microenvironment and is maintained by the self-renewal and differentiation into mature blood lineages of hematopoietic stem and progenitor cells (HSPCs). ID1 is widely expressed in both hematopoietic cells and supportive BM stromal cells, providing regulatory influence over hematopoietic development [16–18]. Notably, many studies have demonstrated that abnormal ID1 expression is strongly correlated with hematologic disorders, including myeloproliferative neoplasms (MPNs), multiple myeloma (MM), and myeloid and lymphoblastic leukemia, positioning it as a potent prognostic biomarker and a viable therapeutic target [19–22]. Herein, we review the ID1 expression profile in the BM hematopoietic system, focusing on its dynamic changes and regulatory roles in lineage commitment and development, and further highlight its crucial roles and therapeutic implications in hematological disorders.

## ID1 expression in HSCs and their immediate progeny

Pluripotent stem cells (PSCs), including blastocyst-derived embryonic stem cells (ESCs) and induced PSCs (iPSCs), possess the pluripotency to differentiate into virtually all cell lineages of an organism, and an unlimited potential for self-renewal and proliferation in the same state. Undifferentiated human ESC (hESC) lines (H1 and H9) and hiPSC line (MchiPSC1.1) show identical *ID1* expression profiles during in vitro hematopoietic induction. *ID1* mRNA level rises rapidly in the hemogenic specification phase (phase I, day 0–7) as assessed by quantitative polymerase chain reaction (qPCR), peaks at the onset of hemogenic precursor emergence on day 7, and subsequently declines in the hematopoietic commitment phase (phase II, day 7–15) [23]. ID1 protein level is high in murine-derived undifferentiated ECSs during embryoid body formation, followed by a transient decrease on the first day of differentiation and returns to baseline on day 5 [24].

**Fig. 2:**
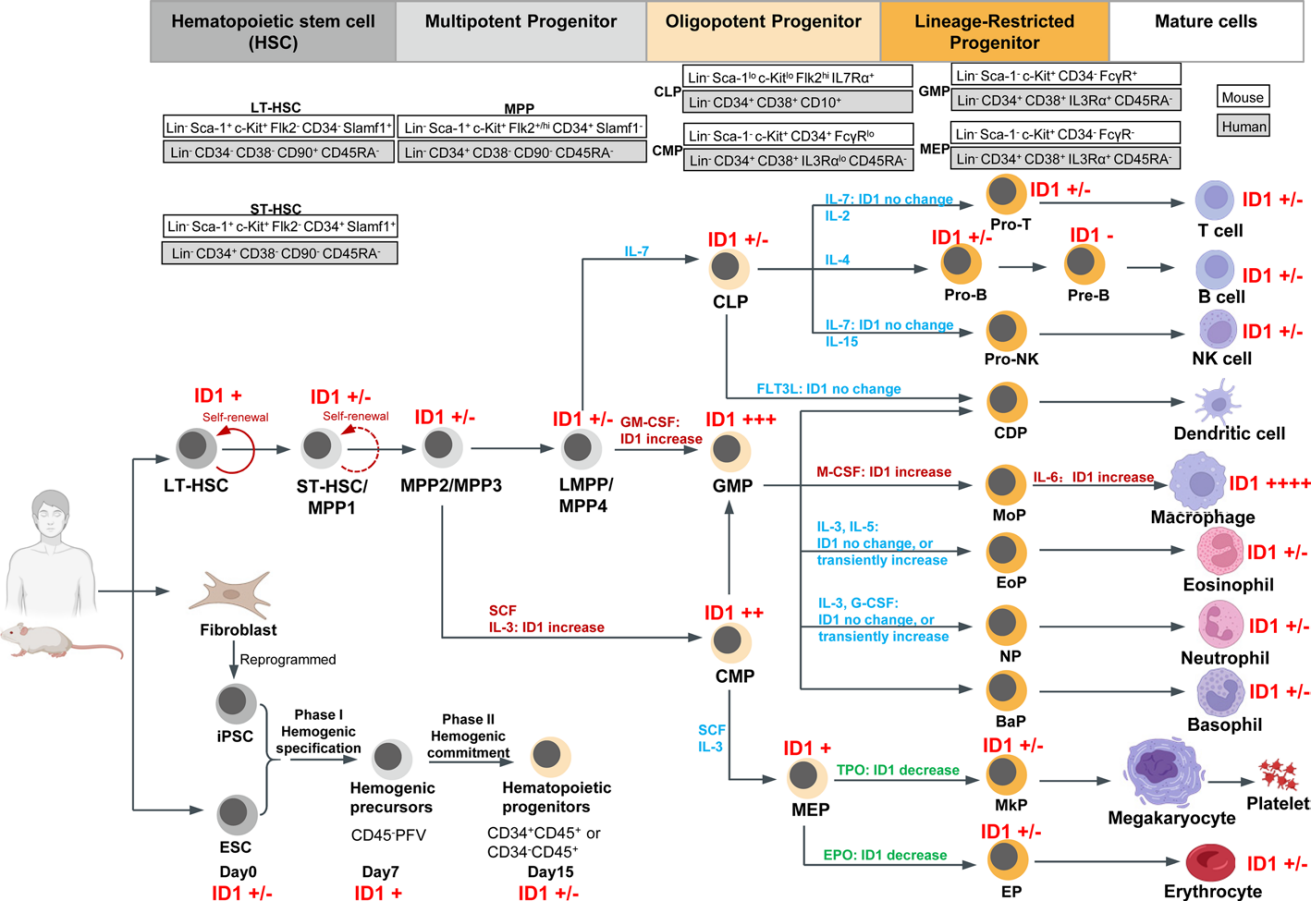
ID1 expression in the classical and revised models of hematopoietic hierarchy. In the classical model of hematopoiesis, long-term HSCs (LT-HSCs), short-term HSCs (ST-HSCs), and multipotent progenitors (MPPs) sit at the upstream of the hematopoietic hierarchy. MPPs differentiate into the branches of the common lymphoid progenitors (CLPs) and common myeloid progenitors (CMPs). The second bifurcation is granulocyte-monocyte progenitors (GMPs) and megakaryocyte erythroid progenitor cells (MEPs), which are divided from CMPs. CLPs generate lymphocytes and dendritic cells, while GMPs give rise to granulocytes, macrophages, and dendritic cells; MEPs produce megakaryocytes/platelets and erythrocytes. In the revised roadmaps of hematopoiesis, lymphoid-primed MPPs with high levels of Flk2 are identified as a subset of the heterogeneous LSK (Lin^−^ Sca-1^−^ c-Kit^+^) progenitor pool. ID1 expression in hematopoietic cells and its changes by cytokine treatment are labeled. Abbreviations: Pro-T, T cell progenitor; Pro-B, B cell progenitor; NK: natural killer; Pro-NK, NK cell progenitor; CDP, dendritic cell progenitor; MoP, monocyte progenitor; EoP, eosinophil progenitor; NP, neutrophil progenitor; BaP, basophil progenitor; MkP, megakaryocyte progenitor; EP, erythroid progenitor; iPSC, induced pluripotent stem cell; ESC, embryonic stem cell; SCF, stem cell factor; IL-3, interleukin 3; G-CSF, granulocyte colony-stimulating factor; M-CSF, macrophage colony-stimulating factor; GM-CSF, granulocyte–macrophage colony-stimulating factor; FLT3L: FMS-related tyrosine kinase 3 ligand; EPO: erythropoietin; TPO: thrombopoietin

HSCs are involved in BM hematopoiesis throughout an individual’s adulthood, providing a lifelong supply of all mature cells needed for hematopoietic and immune systems. The classical hematopoietic hierarchical roadmap is a dogma of hematocyte research, and it demonstrates the stepwise or continuous differentiation process of HSCs into their progeny multilineage progenitors, committed progenitors, and mature lineage cells. In short, HSCs are generally classified into two subpopulations: long-term HSCs (LT-HSCs) and short-term HSCs (ST-HSCs), which are defined as Lin^−^ Sca-1^+^ c-Kit^+^ (LSK) cells (lineage markers, Lin; stem-cell antigen-1, Sca1; KIT Proto-Oncogene, c-Kit). ST-HSCs develop into downstream multipotent progenitors (MPPs), common lymphoid progenitors (CLPs), common myeloid progenitors (CMPs), granulocyte-monocyte progenitors (GMPs), and megakaryocyte erythroid progenitor cells (MEPs). MPP4 is also defined as lymphoid-primed MPPs (LMPPs) with high expression of FMS-related tyrosine kinase 3 (FLT3, also known as *Flk2* in mice). The fate decisions of stem cells are governed by the integrated effects of intrinsic transcription factors and extrinsic cytokines to conduct the stepwise differentiation precisely (Fig. 2) [25–30].

The landscape of ID1 expression profile in all hematopoietic cell lineages and their responses to distinct cytokines important for hematopoiesis has already been charted in vitro and in vivo. A complete *Id1* mRNA expression pattern has been outlined in HSCs and their immediate progeny purified from the normal bone marrow cells (BMCs) derived from C57BL/6 mice. *Id1* transcripts are very low in HSCs and CLPs, markedly increase in CMPs, and further increase in GMPs, but decrease as CMPs mature to MEPs and erythroid cells. *Id1* expression levels are decreased or absent during the differentiation of neutrophil, lymphoid, and erythroid cells, specifically weak or not in TER119^+^ erythroid, Gr-1^+^ granulocyte, B220^+^ B, and CD3^+^ T cells, but remain high in terminally differentiated macrophages [31]. In line with this study, the most primitive LSK^+^ cells in murine BM exhibit higher *Id1* expression than the LSK^−^ subsets, namely oligopotent and lineage-committed progenitors [32]. An *Id1/GFP* knock-in mouse model with a green fluorescent protein (*GFP*) inserted downstream of the *Id1* promoter (*Id1*^*G/G*^) from Sun’s Lab can track and examine ID1 expression on an individual-cell basis of rare BM subsets, as such inserted homozygous mice do not produce ID1 protein. *Id1/GFP* fluorescence is marginally expressed in LT-HSCs, but more abundantly than in ST-HSCs, and LT-HSC activity resides in the *Id1/GFP*-expressing LSK cells [33, 34]. *Id1/GFP* fluorescence is below the detection limit in LMPPs, CMPs, CLPs, MEPs, and CD19^+^ B lineage cells and is expressed only in GMPs. In addition, robust *Id1/GFP* expression has been observed in lineage-positive myeloid cells (Mac-1^+^) and myeloid lineage cells in the blood and peritoneum [34]. Compared with the multipotential erythroid myeloid lymphoid (EML) cell line, the more committed myeloid progenitor (MPRO) cell line has a higher ID1 protein expression level [31]. Thus, the expression pattern of ID1 provides direct evidence supporting the revised model of HSC lineage determination, which proposes that LMPPs generate GMPs and CLPs without transitioning through CMPs. The upregulation of ID1 in GMPs likely contributes to myeloid specification by shutting off the lymphoid option.

Several studies have investigated the dynamic changes of ID1 expression during the lineage differentiation of HSPCs by various hematopoietic growth factors (HGF) stimulation. Interleukin 3 (IL-3), a cytokine that promotes myeloid cell maturation, induces increases in *Id1* transcripts in EML, HSCs, and purified Lin^lo^ c-Kit^+^ Sca-1^−^ BMC progenitor cells supporting myeloid differentiation with or without stem cell factor (SCF). Conversely, IL-7/FLT3 ligand (FLT3L) does not significantly increase *Id1* transcripts in EML, Lin^lo^ BMC, or CLPs during lymphocyte differentiation. In the Lin^lo^ BMC progenitor population, the treatment of macrophage colony-stimulating factor (M-CSF) and granulocyte–macrophage colony-stimulating factor (GM-CSF) elevates *Id1* mRNA levels. However, granulocyte colony-stimulating factor (G-CSF) does not alter *Id1* expression, and erythropoietin (EPO) and thrombopoietin (TPO) significantly down-regulate *Id1* expression [31]. Cytokines promoting myeloid differentiation, such as IL-3, GM-CSF, and IL-6, stimulate robust *Id1* expression in lineage-negative LSK progenitors, but IL-7, IL-4, IL-5, FLT3L, G-CSF, M-CSF, EPO, and TPO show no effects [34]. During eosinophil or neutrophil differentiation from CD34^+^ cells induced by cytokines, ID1 protein transiently increases and declines after extended culture. This pattern suggests that ID1 expression is upregulated during early granulopoiesis and decreases during final maturation [9, 35]. Together, these studies indicate that cytokines favoring myeloid differentiation (e.g., IL-3) can upregulate ID1 expression in multiple HSPCs, thereby directing differentiation toward myeloid cells rather than lymphoid or erythroid cells.

The quantitation of *ID1* gene expression presented here is primarily consistent with studies conducted on hematopoietic cell lines. The summary of ID1 expression in immortalized normal hematopoietic and leukemic cell lines is listed in Table 1. As an antagonist of terminal erythroid differentiation, *Id1* is highly expressed in uninduced murine erythroleukemia cell line MEL, a valuable model to study erythroid differentiation, and decreased when induced to differentiate by chemical inducer dimethyl sulfoxide (DMSO) [3, 36]. In a study analyzing *ID1* expression across 18 human and murine hematopoietic cell lines, *ID1* was detectable in most cell lines but exhibited particularly high expression in several myeloid cell lines, including WEHI-3, NFS60, and FDCP1 [37]. Furthermore, *Id1* mRNA noticeably increases after exposure to IL-3 or GM-CSF in the mouse normal myeloid progenitor cell line FDCP1 and the childhood acute megakaryoblastic leukemia cell line MO7E [38]. In the murine myeloid precursor cell line NFS60, cellular proliferation stimulated by multiple cytokines (SCF, IL-3, L-6, G-CSF, and EPO) positively correlates with *Id1* expression. These findings suggest the cell-cycle regulatory role of *ID1* in multipotent myeloid progenitor cells [39]. In the murine myeloid precursor cell line 32DC13(G), *Id1* mRNA is expressed, but its levels transiently decrease after exposure to G-CSF, which induces terminal differentiation [40]. *ID1* expression is predominantly seen in proliferating bipotential and unipotential progenitors, but is absent in trilineage erythroid-megakaryocyte-macrophage progenitors. Its expression declines along with the differentiation process and increasing maturity of hematopoietic cells, and also with the growth arrest of proliferating cells [37, 38].

**Table 1:** ID1 expression in hematopoietic cell lines

Cell lines	Species	Cell line type	ID1 expression	Refs.	Cell lines	Species	Cell line type	ID1 expression	Refs.
H1; H9	Human	Undifferentiated hESC	Very low (mRNA)	[23]	WEHI-3; NFS60	Murine	Myeloid progenitor leukemia	High level (mRNA)	[37, 39]
MchiPSC1.1	Human	Undifferentiated hiPSC	Very low (mRNA)	[23]	HL60	Human	Myeloid leukemia	Very low (mRNA and protein)	[9, 37]
EML	Murine	Multipotential erythroid myeloid lymphoid	Very low (mRNA)	[31]	THP1; U937	Human	Myeloid leukemia	Very low or expressed; undetectable (mRNA and protein)	[9, 81]
32DC13(G)	Murine	Myeloid precursor	Expressed (transiently decrease after exposure to G-CSF) (protein)	[40]	MOLM13; MOLM14	Human	Myeloid leukemia	Expressed (mRNA and protein)	[22, 81]
MPRO	Murine	Myeloid progenitor	High level (mRNA)	[31]	KG1a	Human	Myeloid leukemia	Very low (protein)	[9]
M1; RAW 246.7	Murine	Monocyte/macrophage	Low (mRNA)	[37]	MC6	Human	Mastocytoma	Very low (mRNA)	[37]
FDCP1	Murine	Myeloid progenitor	High level (increased after exposure to IL-3 or GM-CSF) (mRNA)	[37, 38]	MO7E	Human	Acute megakaryoblastic leukemia (AML M7)	Very low or expressed (increased after exposure to IL-3 or GM-CSF) (mRNA and protein)	[9, 37, 38]
18–81; 22D6	Murine	Pre-B	Very low; low (mRNA)	[3, 37]	MBO2; UT7	—	Acute megakaryoblastic leukemia	Expressed (protein)	[9]
HF-urba	—	B	Expressed (protein)	[9]	Namalwa	Human	Burkitt lymphoma	Very low (mRNA)	[37]
A301; Jurkat	Human	T	Expressed; low (protein)	[9]	P3X; T1165	Murine	Plasmacytoma	Very low (mRNA)	[37]
K562	Human	Erythroleukemia	Very low or high level (mRNA and protein)	[9, 22, 37]	EL4; BW	Murine	Lymphoma precursor T	Very low (mRNA)	[37]
MEL	Murine	Erythroleukemia	Expressed (uninduced status), decreased after exposure to DMSO (mRNA and protein)	[3, 36, 37, 42]	HUT78; CEM-SS; Kit225	Human	T cell lymphoma	Expressed; low; undetectable (protein)	[9]
TF-1	Human	Erythroleukemia	Expressed (protein)	[9]					

hESC: human embryonic stem cell; hiPSC: human induced pluripotent stem cell; AML: acute myeloid leukemia; IL-3: interleukin 3; G-CSF: granulocyte colony-stimulating factor; GM-CSF: granulocyte–macrophage colony-stimulating factor; DMSO: dimethyl sulfoxide

## ID1 affects hematopoietic lineage development and cell-fate decisions

### ID1 negatively regulates the differentiation of ESCs and iPSCs

*Id1*-defective ESCs from *Id1*-knockout mice have a downregulation of the core regulator of self-renewal *Nanog* and downstream upregulation of mesendoderm differentiation marker *Brachyury*. This shift accounts for the impaired self-renewal capacity and increased differentiation propensity. Both forced *Nanog* expression and *Brachyury* reduction are sufficient to restore the self-renewal ability in *Id1*^*−/−*^ ESCs, highlighting the critical role of the *Id1/Nanog/Brachyury* axis in regulating self-renewal and differentiation toward early mesendodermal lineages for ESCs [24]. Knockdown of *ID1* expression by small interfering RNA (siRNA) in hESC or hiPSC-derived human embryos (hEBs) during the early stage (day 0–2) does not affect the total cell number, viability, or hematopoiesis. However, *ID1* silencing during the hematopoietic commitment (day 8–10) substantially augments the output of mature hematopoietic cells from hematopoietic precursors. It increases the colony forming unit (CFU) produced by CD34^+^CD45^+^ hematopoietic progenitors, yet does not alter CFU subtype distribution [23]. Constitutively expressing *ID1* in the hESCs has inhibited the activity of E-proteins necessary for lymphocyte development and failed to produce T and B cells [41]. Consequently, during the hematopoietic commitment phase, the negative regulatory role of ID1 on hESC and hiPSC directly affects hematopoietic precursors.

### ID1 effects on hematopoiesis in *Id1*-overexpressed models

ID1 overexpression can facilitate cell fate determination of myeloid versus lymphoid lineage commitment in HSCs, myeloid versus erythroid cell development in CMPs, and macrophage versus granulocyte in committed GMPs. *Id1* overexpression in HSCs and the multipotential clonal stem cell line EML impairs erythroid and B cell differentiation in vitro and in vivo. Although the high level of *Id1* does not affect the early stages of myeloid development, it inhibits the final stages of granulocyte but not macrophage differentiation [31]. Specifically, the murine erythroleukemia MEL cell line that constitutively expressed functional ID1 protein shows inhibited DMSO-induced terminal erythroid differentiation, along with blocked activation and expression of certain erythroid-specific genes [36, 42]. The constitutive expression of *Id1* in CD34^+^ cells inhibits eosinophil proliferation and development while modestly enhancing neutrophil differentiation in vivo and in vitro, suggesting that *Id1* deficiency is seemingly required for eosinophil differentiation but not neutrophil maturation [35]. Forced expression of *Id1* in LSK progenitors gives rise to largely Mac-1^+^ myeloid cells and very few CD19^+^ B cells. Consistently, *Id1*-expressing LSK progenitors in the transplantation model produce a greater percentage and number of myeloid cells while significantly decreasing lymphoid lineage cells [34]. Moreover, *Id1* overexpression in BMCs enhances the proliferation and expansion of primitive progenitors and immature myeloid cells [9]. Mechanistically, E-protein activity enhancement can counteract ID1-mediated inhibition of E-proteins to prevent the myeloid cell fate and drive B cell differentiation. This observation underscores the crucial role of the coordination between E protein transcription factors and their inhibitor, ID1 protein, for determining the myeloid-versus-lymphoid fate choice [34]. Furthermore, the direct interplay between transcription factors growth factor independent 1 transcriptional repressor *(Gfi1)*, *Tcf3*, and *Id1* is a key element in determining the fate of progenitor cells between myeloid and lymphoid lineages. High levels of *Gfi1* in multipotent progenitors can repress *Id1* and potentiate the positive transcriptional activity of *Tcf3*, on its downstream B cell specific genes to commit towards B lymphoid lineage and block myeloid differentiation [43, 44]. Thus, these studies supported that ID1 expression drives myeloid development at the expense of lymphoid formation.

### Evaluation of ID1 effects on hematopoietic system in *Id1*-deficient models

In addition, several studies comprehensively evaluated the changes in the hematopoietic system in *Id1*-deficient mouse models. Jankovic et al. reported for the first time that *Id1*-null mice harbor normal myeloid cell numbers, slightly decreased erythrocytes, and significantly decreased circulating lymphocytes in peripheral blood (PB). Both *Id1*^*−/−*^ LSK^+^ and *Id1*^*−/−*^ LSK^−^ cells show an increased tendency of premature myeloid commitment and cell cycle entry, along with a premature loss of self-renewal capacity under conditions of replicative stress. *Id1* overexpression can reverse the premature commitment to myeloid differentiation in *Id1*^*−/−*^ LSK^+^ cells, but not *Id1*^*−/−*^ LSK^−^ cells, indicating that ID1 acts as a specific differentiation inhibitor to control the early stages of HSCs commitment. In *Id1*^*−/−*^ BM, the MEP/GMP ratio is increased along with inappropriate expression of myeloerythroid transcription factors, indicating that the absence of ID1 in vivo favors myeloerythroid lineage commitment [32], which is consistent with ID1 as an antagonist for erythroid differentiation [36, 42, 45]. Similarly, *Id1*-deficient mice also exhibit normal steady-state hematopoiesis. *Id1*^*−/−*^ grafts do not appear to diminish primary engraftment, but have impaired secondary transplantation potential with poorly reconstituted BM and PB. *Id1* deficiency gives rise to impaired function and a two-fold reduced number of LT-HSCs, which may be mechanistically due to the promotion of *Id1* deficiency on E-proteins to activate the cyclin-dependent kinase inhibitors *p21* and *p16* transcriptionally, and subsequently reduce LT-HSCs expansion, numbers, and engraftment potential [33].

**Fig. 3:**
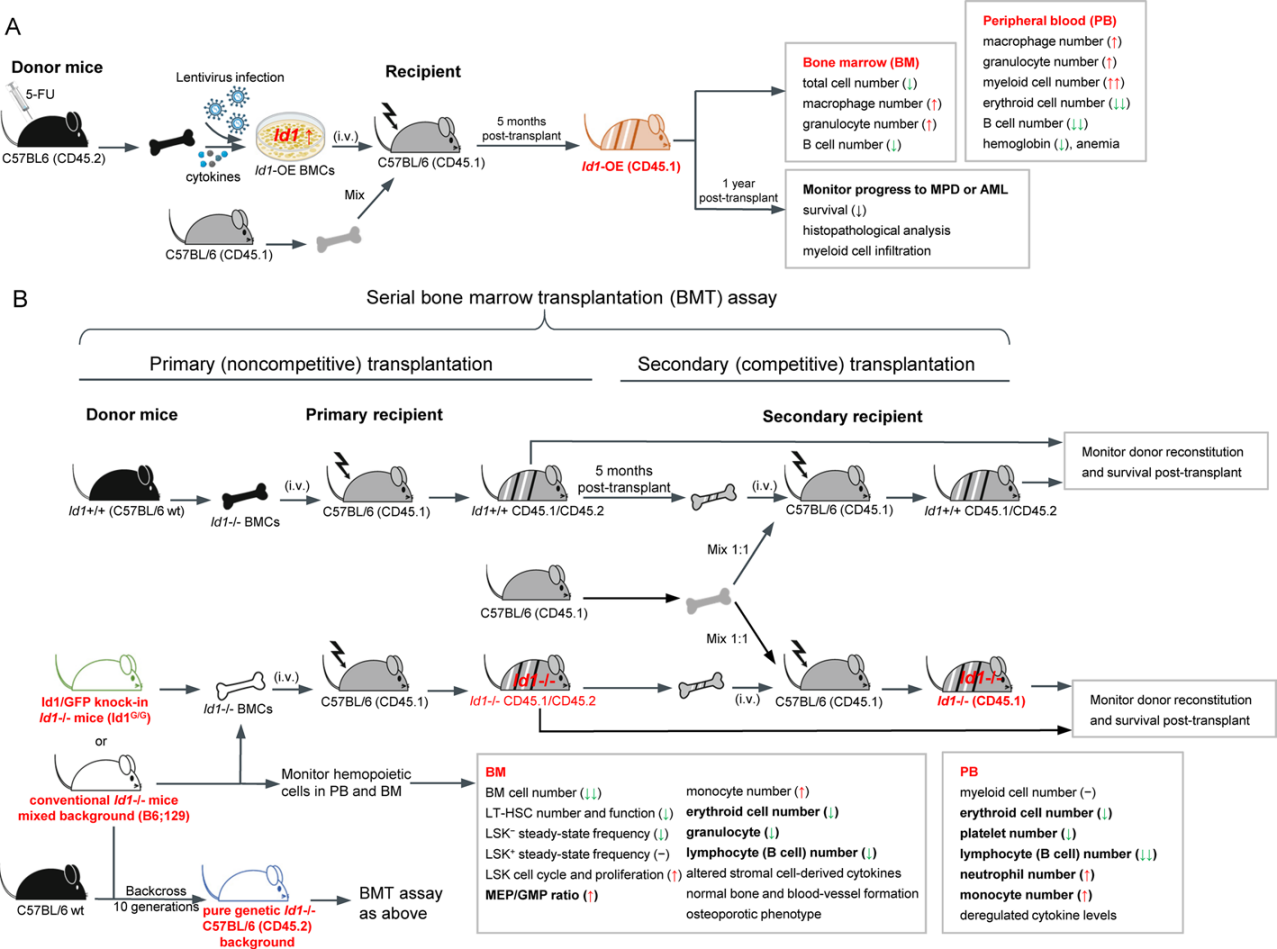
Schematic diagram and experimental design of whole *Id1*-deficient and *Id1*-overexpressed mouse models for studying the effects of ID1 on hematopoiesis. **A** 5-fluorouracil (5FU)-treated C57BL/6 (CD45.2) mice-derived bone marrow cells (BMCs) are infected with *Id1*-overexpression lentivirus or vector control, and then injected into the tail vein of irradiated C57BL/6 (CD45.1) mice with support marrow from CD45.1 mice. Several months after transplantation, hematopoietic cells in peripheral blood (PB), BM, spleen, and thymus are analyzed to evaluate the hematopoietic reconstitution and survival of *Id1*-OE mice, summarized from [9, 31, 34]. **B** The *Id1*^*−/−*^ mice on a conventional mixed background [17, 32, 46], pure genetic background [47], or *Id1/GFP* knock-in [33], or *Id1*^+*/*+^ C57BL/6 wild-type, acts as a donor to provide BMCs to transplant into the irradiated primary recipient, respectively. Several months after transplantation, CD45.2 donor cells, and equal numbers of CD45.1 competitors, are transplanted into secondary recipients. The hematopoietic cells and hematopoietic reconstitution are monitored in *Id1*^*−/−*^ mice and primary/secondary recipients. The green arrow indicates a decrease, the red arrow represents an increase, and (-) represents unchanged levels. GFP: green fluorescent protein

Another study independently examined the impaired hematopoiesis in the same *Id1*^*−/−*^ mice. Also, it demonstrated the essential role of ID1 in maintaining the tri-lineage development and hematopoietic progenitor cell niche in the BM. The total nucleated BMCs is in *Id1*^*−/−*^ mice is substantially reduced. *Id1*^*−/−*^ BM shows an increased percentage of CD34^lo^ LSK, LSK, and CMP but normal absolute numbers and self-renewal of HSCs, suggesting that *Id1* is not required for HSCs self-renewal or maintenance [17]. Another study reported that the BM CMPs subpopulation decreases, MEPs and CD11b^+^ monocyte subsets expand, and peripheral fraction of monocytes and neutrophils increases in *Id1*^*−/−*^ mice. Moreover, in steady-state and hematopoietic stress conditions, *Id1*^*−/−*^ mice have more *Id1*^*−/−*^ LSK cells entering cell cycle, with enhanced proliferation and increased cell numbers [46]. Further backcrossing the conventional *Id1*^*−/−*^ mixed background mice (B6;129) with C57BL/6 mice for ten generations revealed that the hematopoietic injury induced by the loss of *Id1* is less severe on the pure genetic C57BL/6 background compared with the mixed background. In the stress hematopoiesis of chronic inflammation and aging mimicked by the serial bone marrow transplantation (BMT) assay, genetic ablation of *Id1* enhances HSC self-renewal and protects HSCs from exhaustion. After BM transplantation, *Id1*^*−/−*^ HSCs show a quiescent phenotype and molecular signature, including reduced proliferation, cell cycle, mitochondrial biogenesis/stress, and oxidative stress regulated by the *Id1-Tcf3-p16* pathway [47]. Intriguingly, a series of BMT experiments from these independent studies generally demonstrates that *Id1*^+*/*+^ recipient mice transplanted with *Id1*^*−/−*^ HSCs have normal hematopoietic development but exhibit reduced survival. However, *Id1*^*−/−*^ recipients with *Id1*^+*/*+^ HSCs transplantation show increased survival but still reproduce the impaired hematopoiesis of *Id1*^*−/−*^ mice. These studies indicate that *Id1* operates through both intrinsic and extrinsic signals in the BM niche. Here, we comprehensively review the changes in the hematopoietic system in *Id1-*knockout models, including the *Id1*^−/−^ mice on conventional mixed background [17, 32, 46], pure genetic background [47] and *Id1/GFP* knock-in [33] act as a donor to provide BMCs. The application of different experimental models and detection methods may be the primary reason for differences in the findings of these studies (summarized in Table 2 and Fig. 3). Overall, we can conclude that the *Id1*-deficient BM microenvironment cannot support normal hematopoietic development and cannot be rescued entirely by wild-type BM [9, 17, 46, 47].

**Table 2:** The strategies of studying ID1 expression and function in hematopoiesis using different experimental models

ID1 expression		ID1 function		Main conclusion	Refs.
Cell	Mice	Cell	Mice		
*Id1*-overexpressed models					
1. EML, MPRO (by Microarray, Northern/WB) 2. EML/BMC/Lin^lo^ c-Kit^+^ Sca-1^−^ BMC induced with cytokines (by qPCR or WB)	BMCs obtained from C57BL/6 mice and sorted by FCM	*Id1*-overexpressed EML induced with cytokines	BMCs from 5FU-treated C57BL/6 (CD45.2) mice infected to overexpress *Id1* and then transplanted into irradiated C57BL/6 (CD45.1) mice with support marrow from CD45.1 mice. 4–6 months after transplantation, evaluate hematopoietic reconstitution by analyzing PB, BM, spleen and thymus using FCM	1. Obtain complete ID1 expression pattern in HSCs and immediate progeny by qPCR 2. ID1 facilitates cell fate of myeloid versus lymphoid, myeloid versus erythroid, and macrophage versus granulocyte lineage commitment	[31]
Pre-B cell line (18–81), erythroleukemia cell line (MEL) (by densitometic scanning of autoradiograms or Northern blot)	NA	Constitutive expression of *Id1* in MEL induced with chemical inducer DMSO	NA	ID1 inhibits terminal erythroid differentiation	[3, 36, 42]
LKS cells induced with a set of cytokines (by GFP fluorescence)	BMCs analyzed and sorted in *Id1/GFP* knock-in mice (*Id1*^G/G^)	*Id1*-overexpressed LKS cells induced with cytokines	As described in [31]	1. Obtain complete ID1 expression pattern in HSCs and immediate progeny by fluorescence 2. ID1 facilitates cell fate of myeloid versus lymphoid lineage commitment	[34]
1. Several murine and human hematopoietic cell lines, AML cell lines, and BMCs from patients with MDS/AML and healthy donors (by WB) 2. CD34^+^ BMCs from healthy donors induced with cytokines (by WB)	NA	1. BMCs from 5FU-treated C57BL/6 (CD45.2) mice infected to overexpress *Id1* (by GFP fluorescence) 2. *Id1* silenced in AML cell line MO7E by siRNA and detect cell growth	As described in [31] and then evaluate one year survival	1. ID1 expressions in various cell lines 2. ID1 immortalizes hematopoietic progenitors and promotes MPNs and AML	[9]
CD34^+^ cells isolated from umbilical cord blood induced with cytokines (by qPCR and WB)	NA	*Id1*-overexpressed CD34^+^ cells induced with cytokines (by GFP fluorescence)	*Id1*-overexpressed CD34^+^ cells transplanted into irradiated NOD/SCID mice. 6 months after transplantation, evaluate eosinophils, and neutrophils of human BM cells	NA	[35]
NA	NA	NA	Constitutive expression of *Id1* in transgenic mice and then detect B cell development	Constitutive expression of ID1 impairs B cell development	[49]
NA	NA	NA	*Id1* cDNA is driven by a T cell-specific proximal promoter of the lck gene. *Id1* transgenic fragment microinjected into FVB/N mice to generate T cell-specific *Id1* transgenic mice	ID1 specifically expressed in T cells leads to T cell deficiency, apoptosis and developmental block	[50]
NA	NA	NA	T cell-specific *Id1* transgenic mice as described in [50] crossed with *Rag1*^−/−^ mice	ID1 specifical expression exacerbates thymocytes loss and apoptosis	[51]
NA	NA	*Id1*-overexpressed T cell lymphoma cell line 16610D9	As described in [50]	ID1 stimulate NF-κB and T cell developmental defects in vitro and in vivo	[52]
NA	NA	1. Naïve CD4^+^ T cells isolated from CD4-*Id1* transgenic mice response on anti-CD3 stimulation with or without CD28 costimulatory signaling 2. Treg cell induction and differentiation from CD4-*Id1* transgenic mice with or without CD28 costimulatory	*Id1* cDNA is driven by CD4 promoter and injected into FVB/N mice to generate CD4-*Id1* transgenic mice	ID1 expression promotes peripheral CD4^+^ T and Treg cell proliferation	[53, 54]
*Id1*-deficient models					
NA	LKS cells isolated and analyzed by FCM	*Id1*-overexpressed *Id1*^*−/−*^ mice-derived LKS^+^ and LKS^−^ cells induced with cytokines	Serial BMT assay conducted as below: 1. Primary (noncompetitive) transplantation: BMCs from *Id1*^+*/*+^ (C57BL6 wt) or conventional *Id1*^*−/−*^ (CD45.2) mice transplanted into irradiated B6.SJL (CD45.1) mice 2. Secondary competitive transplantation: 5 months after primary transplantation, *Id1*^*−/−*^ (CD45.2) donor cells together with equal numbers of B6.SJL (CD45.1) competitors transplanted into secondary recipients	1. LKS^+^ cells have higher ID1 expression than LKS^−^cells 2. Identify quantitative and qualitative defects in the HSC pool of *Id1*-deficient mice	[32]
NA	BMCs analyzed and sorted in *Id1/GFP* knock-in mice (*Id1*^G/G^)	NA	BMT assay in *Id1*^*G/G*^ mice conducted as described in [32]	1. LT-HSCs have more abundant ID1 expression than ST-HSCs 2. *Id1* deficiency has impaired LT-HSCs and secondary transplantation	[33]
NA	NA	NA	As described in [32], and also detect the cytokines/chemokines in stromal cells and serum	1. *Id1* deficiency mice have impaired hematopoiesis and cannot support normal hematopoietic development 2. *Id1* deficiency stromal cells have altered cytokines production to regulate hematopoietic progenitor cell niche	[17]
NA	NA	LSK cells isolated from *Id1*^*−/−*^ mice, and detect cell cycle and proliferation	As described in [32], and also assess the osteoporotic phenotype	1. *Id1* deficiency mice have osteoporotic phenotype 2. ID1 is a principal player responsible for the cross-talk between bone and BM hematopoietic cells	[46]
NA	NA	NA	Conventional *Id1*^*−/−*^ mixed background mice (B6; 129) backcross with C57BL/6 mice for 10 generations to pure genetic *Id1*^*−/−*^ mice on C57BL/6 background, and BMT assay conducted as described in Jankovic et al (2007) [32]	*Id1*^*−/−*^ HSCs show a quiescent phenotype and molecular signature to protect from exhaustion	[47]
NA	BMCs analyzed and sorted in *Id1/GFP* knock-in mice (*Id1*^G/G^)	NA	*Id1*^G/G^ mice conducted primary transplantation and then focus on B cell differentiation	ID1 ablation enhances B-lineage cell production and differentiation	[48]
Spleen-derived naïve CD4^+^ T cells induced with under subset-polarizing conditions to detect *Id1* expression in Th1, Th2, Th9, Th17 and Treg cells (by qPCR)	NA	Spleen-derived naïve CD4^+^ T cells transduced with shRNA or sgRNA to knockdown *Id1*; or transduced with *Id1* expressing retroviral vector to overexpress *Id1*	1. Conventional *Id1*^*−/−*^ (CD45.2) mice as described in Jankovic et al. (2007) [32], and assess Th9 differentiation 2. *Id1*^*fl/fl*^ mice cross with *Cd4*^*cre*^ mice to obtain *Id1*^*fl/*+^*-Cd4*^*cre*^ (*Id1* hetero) mice and *Id1*^*fl/fl*^*‐Cd4*^*cre*^ (*Id1* cKO) mice, and assess Th9 differentiation	ID1 is an essential positive regulator of Th9 cell differentiation	[55]

qPCR: quantitative polymerase chain reaction; WB: western blot; FCM: flow cytometry; BMT: bone marrow transplantation; siRNA: small interfering RNA; shRNA: short hairpin RNA; sgRNA: single guide RNA; MPN: myeloproliferative neoplasm

### ID1 regulates lymphocyte development

Although *Id1*-deficient mice (*Id1*^*G/G*^) do not exhibit significant abnormalities in steady-state B lymphopoiesis, the transplant recipients of *Id1*-deficient BM show more robust B cell engraftment without changes in myeloid differentiation. Nevertheless, this advantage in B cell differentiation is quickly masked by the dominant balanced capacity of the hematopoietic system five weeks post-transplantation. *Id1* deficiency promotes the generation of B lineage cells and enhances the intrinsic ability to differentiate along B lineage in the LSK multipotent progenitors when cultured with cytokines SCF, FLT3L, and IL-7. However, the increased B lymphopoiesis is not at the expense of myeloid lineage cells. *Id1*^*G/G*^ mice exhibit detectable ID1 protein and mRNA expression in pro-B (B220^+^CD43^+^) but not pre-B cell (B220^+^CD43^−^) compartments [48]. Constitutive expression of *Id1* in transgenic mice blocks the B cell maturation process of the transition from pro-B to pre-B cells, inhibits V(D)J and VκJκ recombination frequencies of the immunoglobulin loci, and reduces B cell-specific gene expression [49]. Thus, ID1 deficiency promotes the generation and differentiation of B lineage cells.

The T cell-specific *Id1* transgenic mouse line exhibits a dramatic reduction in the total number of thymocytes, and most of them are severely blocked at the earliest progenitor stage of CD4 and CD8 double negative (DN) [50]. During normal thymus T cell development, the T cell receptor (TCR) signal leads to a balanced activation of the NF-κB and the bHLH transcription factors (*Tcf3* and *Tcf12*) to ensure proper differentiation and proliferation. When the *Id1* transgenic mouse is further crossed with *Rag1*-deficient mice with TCR signaling absence, it exhibits a strongly potentiated NF-κB signal by *Id1* expression, allowing T cells to differentiate from DN to double positive (DP) stage [51]. Subsequent in vitro assays prove that ID1 expression or E-protein inhibition directly stimulates NF-κB and its target cytokines, including tumor necrosis factor-α (TNF-α), in both DP T cell lymphoma cell line 16610D9 and thymocytes isolated from *Id1* transgenic mice [52]. Once cells reach the DP stage, *Id1* overexpression induces massive apoptosis of differentiating T cells by up to 50% of the total thymocytes, probably through effectively inhibiting the expression of the E-protein-activated antiapoptotic genes. Thus, *Id1* plays an oncogenic role in developing T cell lymphoma in adult transgenic mice at a high frequency [50]. However, CD4 promoter-driven *Id1* transgenic mice have a healthy life with normal T cell development in the thymus and periphery. Ectopic *Id1* expression can promote robust anti-CD3-induced proliferation and survival of peripheral naïve CD4^+^ T cells, and stimulate NF-κB activation and IL-2 production in the absence of CD28-mediated TCR costimulation. As mice aged, compared with wild-type mice, *Id1* transgenic expression facilitates thymocyte differentiation into T regulatory (Treg) lineage without CD28 costimulatory signaling, resulting in elevated thymic Treg cell output and increased peripheral Treg cell counts. The increased level of Treg cells leads to enhanced immunosuppressive function and reduced susceptibility to autoimmune diseases in *Id1* transgenic mice [53, 54]. Among the various T helper subsets induced by subset-polarizing conditions of spleen-derived naïve CD4^+^ T cells from wild-type mice, *Id1* is specifically expressed in Th9 cells compared with Th1, Th2, Th17, and Treg cells. Under physiological conditions, the expression of *Id1* induced by IL-4 and TGF-β in naïve CD4^+^ T cells can transcriptionally inhibit *Tcf3* and *Tcf4* to enhance IL-9 production and Th9 differentiation. In line with the idea that *Id1* is an essential positive regulator of Th9 cell differentiation, both *Id1*-deficient naïve CD4^+^ T cells from CD4 T cell-specific *Id1*-deficient and whole *Id1*-knockout (KO) mice are unable to differentiate into Th9 cells or produce IL-9. Conversely, *Id1* overexpression enhances IL-9 expression and Th9 cell differentiation. Naïve CD4^+^ T cells from different degrees of *Id1*-deficient models prove that *Id1* expression has a gene dosage effect on IL-9 production; that is, IL-9 expression depends on *Id1* levels. *Id1*-deficient Th9 cells express increased genes related to Type I immune responses and can ameliorate airway inflammation in asthma mouse models [55]. Therefore, it is clear that ID1 expression enhances the production and differentiation of Th9 cells.

### Strategies for studying ID1 expression and function in hematopoiesis

**Fig. 4:**
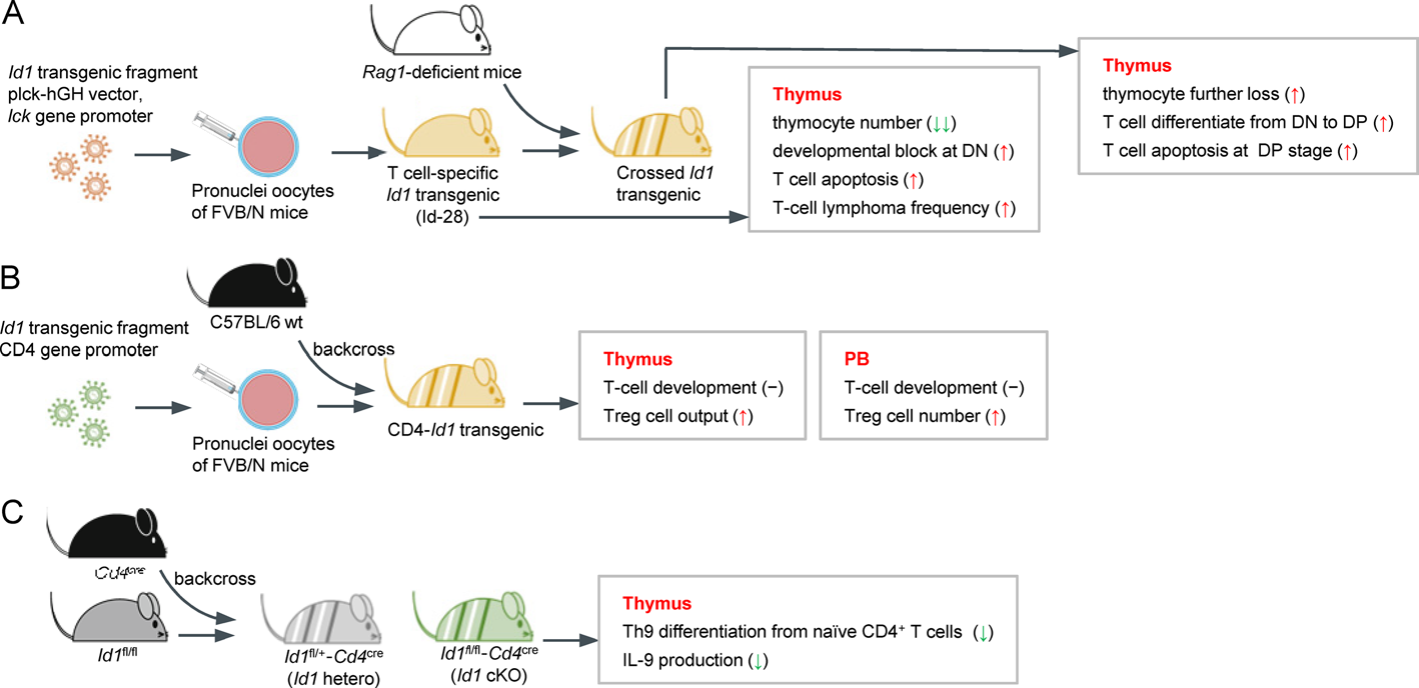
Schematic drawing of *Id1* conditional mouse model to evaluate ID1 roles in specific hematopoietic cell lineages. **A** T cell-specific *Id1* transgenic mice in homozygous background [50, 52] and the heterozygous transgenic mice by crossing with *Rag1*-deficient mice [51] for studying the effects of *Id1* on thymus T cell development. **B** CD4-*Id1* transgenic mice generated by inserting *Id1* cDNA into a CD4 transgenic vector are used to study T cell developmental profiles in the thymus and periphery, especially regulatory T cell (Treg) [53, 54]. **C** *Id1* hetero mice and CD4^+^ T cell-specific *Id1*-deficient mice for studying the differentiation of T cell subsets, especially Th9 [55]

Understanding the ID1 expression profile and its roles in the hematopoietic system depends on formulating rational and feasible strategies. Various research models and detection methods may explain the differences in findings observed in some studies. Thus, we have compiled the strategies from the published literature, which can be implemented independently or in conjunction (Table 2, Figs. 3 and 4). Three strategies can be employed when studying the expression of ID1: First, on the basis of the expression pattern of surface markers, BM, PB, spleen, and thymus samples from healthy mice are sorted and analyzed by flow cytometry. Both protein and transcription levels of ID1 in various purified hematopoietic lineage subsets can be detected. This method is simple and effective for acquiring ID1 expression in the hematopoietic system [31, 32]. Second, *Id1* knock-in mice (*Id1*^*G/G*^), in which *GFP* is inserted downstream of the *Id1* transcriptional start site, can be used to trace and obtain *Id1* expression on an individual cell level with high-resolution information [33, 34, 48]. Third, immortalized human and murine HSPC lines or freshly isolated primary cells are stimulated to differentiate by treating them with a set of cytokines important for hematopoiesis, and ID1 expression and cell differentiation are detected, which can mimic the response of ID1 to differentiation signals [31, 34, 35]. To study ID1 function, three key experimental strategies are commonly employed. First, up- and downregulating ID1 on immortalized or primary HSPCs can be used to evaluate the effects of ID1 on hematopoietic cell differentiation [9, 31, 32, 34, 35, 52, 55]. Second, the number and function of hematopoietic cells in the BM, PB, spleen, and thymus samples from *Id1*-overexpressed or -deficient mice (including whole KO and conditional deletion mice) are compared with control mice [31–35, 48–55]. Third, serial bone marrow transplantation (BMT) assays are performed using *Id1*-deficient mice to evaluate hematopoietic reconstitution capacity and survival [32, 33, 48].

## ID1 expression and roles in stromal cells of the bone marrow niche

Besides the expression in hematopoietic cells, ID1 is widely expressed in other stromal cells to regulate cellular proliferation and differentiation, and hematopoietic homeostasis in the BM niche through a cell-nonautonomous mechanism [17, 18]. The absence of *Id1* does not affect bone or blood-vessel formation in the hematopoietic microenvironment. However, multiple cytokines and chemokines are altered in vitro and in vivo in *Id1*^*−/−*^ mice, which may partially account for the impaired hematopoietic phenotypes in *Id1*^*−/−*^ mice. *Id1*^*−/−*^ stromal cells produce increased levels of G-CSF and GM-CSF but decreased levels of SCF, M-CSF, osteopontin, fibroblast growth factor-1, transforming growth factor-α, and stromal cell-derived factor-1α in vitro. *Id1*^*−/−*^ mice also display decreased serum concentrations of IL-6, TNF-α, VEGF, GM-CSF, SCF, and M-CSF. However, the causal relationship between altered cytokines/chemokines production and hematopoietic defects remains incompletely understood [17]. On the other hand, another study suggested that the disordered homeostasis of myeloid precursor cells increases the number, differentiation, and resorption activity of osteoclasts, ultimately resulting in the osteoporotic phenotype of low bone mass and increased bone fragility. *Id1*-deficient osteoclasts have increased expression of key genes (e.g., the gene coding a proteolytic enzyme secreted by osteoclasts, *Ctsk*) necessary for osteoclast maturation, identifying ID1 as a prime regulator in the cross-talk between bone and BM hematopoietic cells. Mechanistically, the increased secretion of CTSK in the BM microenvironment can cleave cytokines that regulate the proliferation, survival, and mobilization of HSPCs [46].

The differentiation of hESC provides an abundant source of endothelial cells for vasculogenic development. ID1 plays a critical role in regulating angiogenesis and vascularization, as well as maintaining the cellular expansion and commitment of endothelial cells derived from hESCs [56, 57]. Hence, the enhanced hematopoietic potency observed in the *Id1*-deficient hemogenic precursors originating from hemogenic endothelial precursors may be an indirect effect of endothelial development inhibition [23]. In mice with endothelial cell-selective *Id1*-deficiency, the BM sinusoids exhibit progressive deterioration, characterized by loss of integrity, significant dilation, leakage, collapse, and a pro-inflammatory state. Mechanistically, *Id1* gene ablation contributes to activating cell-cycle inhibitors (*p21* and *p27*) by E-proteins, coupled with reduced apoptotic *Bcl2*-family gene expression, resulting in increased apoptosis and suppressed proliferation in sinusoidal endothelial cells. The disruption of sinusoidal integrity and neovascularization leads to a gradual decline in hematopoietic function, marked by increased premature activation, proliferation, differentiation, migration, and exhaustion of HSCs. Therefore, ID1 is intrinsically required for the steady-state survival and regeneration of sinusoidal endothelial cells to maintain proper vessel permeability and hematopoietic homeostasis [58].

## ID1 and hematologic disorders, particularly leukemia

### ID1 is an unfavorable prognostic factor for leukemia

*ID1* has been identified as a potential proto-oncogene in leukemia. However, the expression levels and prognostic values of *ID1* remain controversial and inconclusive owing to different cohorts and statistical methods. Compared with healthy controls, elevated *ID1* expression has been observed in BMCs of acute myeloid leukemia (AML) and myelodysplastic syndrome (MDS) patients [9, 18, 59]. *ID1* is detectable and expressed in about 20% (50/285) of patients with primary AML and is unrelated to the French-American-British (FAB) subtypes, but it is higher in patients with -5/7(q) and t(15;17) [9]. High *ID1* expression is also significantly associated with older age and FLT3/ITD^+^ [9, 59, 60]. Tang et al. analyzed *ID1* expression and its possible prognostic value in 237 patients with AML for the first time [60]. The studies revealed a striking correlation between high *ID1* expression and shorter overall survival (OS) and disease-free survival (DFS) in total patients with AML, as well as in specific subtypes such as M2 and M5 [18, 59, 60]. More importantly, our study demonstrated that *ID1* is an independent unfavorable prognostic factor for 173 patients with AML from the Cancer Genome Atlas public database [61]. High *ID1* expression is an independent risk factor for young non-M3 patients, but not for the whole AML, in our cohort of 102 de novo Chinese patients with AML [59]. Thus, cytogenetically normal (CN) young patients with high *ID1* expression have lower complete remission (CR) rates, and worse OS and DFS, suggesting that *ID1-*negative patients should be classified as unfavorable-risk leukemia [18, 59–61]. In another large cohort of 269 young patients with CN-AML, *ID1* acts as an independent negative prognostic factor when the mutational status of CCAAT/enhancer-binding protein-alpha (CEBPA) is not considered. However, *ID1* loses its prognostic impact when CEBPA mutation is considered in the multivariate analysis [62]. CEBPA is a transcription factor essential for normal differentiation of myeloid progenitors, and *ID1* has been identified as a critical direct target gene of CEBPA [45, 63, 64]. An exome sequencing analysis of a cohort of 263 patients with AML revealed that *ID1* gene mutations are rare in AML, with a detection rate of only 2.66%. *ID1* mutations are more common in cytogenetically normal patients with four novel nonsynonymous mutations (G40C, A124G, A230G, and A349G), and another new mutation, A290G, observed in a case with 11q23 deletion. Among them, the algorithm predicts that four mutations (G40C, A124G, A230G, and A290G) likely alter ID1 protein function [65]. Studies involving Colombian and Hispanic adult patients newly diagnosed with B cell acute lymphoblastic leukemia (B-ALL) indicate that high expression of *ID1* may predict an inadequate response to induction treatment and a poor prognosis [66, 67]. Collectively, it is reasonable to speculate that aberrant high *ID1* expression is correlated with adverse clinical outcome, and may be a surrogate consequence of other genetic variants, rather than a primary genetic event of leukemogenesis [63].

### The therapeutic potential of ID1 in AML

ID1 is widely expressed in most AML cell lines, particularly in myeloid cell lines. Silencing *ID1* using siRNA in the AML cell line MO7E inhibits cell growth, suggesting that *ID1* is required to maintain leukemia cell hyperproliferation [9]. Our research has revealed that the downregulated miR-29b in the demethylating drug decitabine (DAC)-resistant chronic myeloid leukemia cell line (K562/DAC) leads to abnormal upregulation of *ID1* and resistance to apoptosis. This finding indicates that *ID1* is involved in the mechanism by which hypomethylating agents induce oncogene upregulation and secondary drug resistance [68, 69]. However, some studies have shown contradictory findings, indicating that *ID1* expression is barely detectable in most AML cell lines and patient samples. The histone deacetylase inhibitor trecomycin A alone or combined with DAC can restore *ID1* expression and induce more cell apoptosis and death [70]. All-trans retinoic acid can achieve therapeutic effects in acute promyelocytic leukemia (APL) cell lines and primary patient cells by rapidly increasing *ID1* expression and downregulating TCF3, leading to G0/G1 cell-cycle arrest [71].

The aberrantly activated oncogenic tyrosine kinases and their downstream overlapped signaling pathways play important roles in the pathogenesis of hematopoietic malignancies [72]. Using a comparative gene expression strategy, ID1 is identified as a common downstream target of various oncogenic tyrosine kinases. The AML cell line MOLM14 and chronic myeloid leukemia cell line K562, which express FLT3-ITD and BCR::ABL1, respectively, exhibit growth inhibition, increased p27^Kip1^ expression, and enhanced apoptosis upon the knockdown of *ID1*. Mechanistically, the transcriptional target of FLT3-ITD tyrosine kinase on *ID1* may depend on PI3K/AKt or JAK-STAT signaling cascades [22]. ID1 is required for BCR::ABL1-mediated leukemogenesis, and its expression is regulated by BCR::ABL1-STAT5 pathway to enhance the invasiveness of leukemia cells mediated by matrix metalloproteinase 9 [73]. Furthermore, the constitutively activated PI3K/PKB in BCR::ABL1-transformed cells can phosphorylate and inactivate FOXO3a, and abrogate its direct transcriptional repression of *ID1* to maintain the leukemic phenotype [74]. The transcriptional coactivator p300 acetylates AML-ETO oncogenic fusion protein generated by the translocation of the t(8;21). This acetylation allows p300 to co-occupy the *ID1* promoter with AML-ETO, thereby inducing t(8;21) leukemia [75]. *Id1* deletion can abrogate and slow down leukemia initiation, and markedly prolong the survival time of the exon9a isoform of AML-ETO (AE9a) mice. Conversely, *Id1* overexpression promotes the self-renewal of leukemic stem cells (LSCs) and accelerates leukemogenesis. Cannabidiol (CBD), an ID1 inhibitor, downregulates ID1 mRNA and protein levels dose-dependently. CBD can inhibit the downstream AKT1/mTOR signaling, leading to cell-cycle arrest and apoptosis to block AE9a-driven leukemogenesis and prolong survival. CBD also sensitizes AML cells to AKT inhibitors and shows synergistic antileukemic effects when combined with AKT inhibitors, whereas normal hematopoietic cells are spared [18, 76, 77].

ID1 is usually polyubiquitinated and rapidly degraded by the proteasome. USP1, the most well-studied deubiquitinase, can deubiquitinate and stabilize ID1 protein by removing the polyubiquitin chains from ID1 to prevent degradation. When ectopically expressed in MSCs, USP1 stabilizes ID1 protein, blocks the normal osteogenic differentiation program, and induces proliferation to promote tumorigenesis. USP1 short hairpin RNA (shRNA) can rapidly degrade ID1, induce p21-mediated cell-cycle arrest, and initiate an osteogenic differentiation program in osteosarcoma, providing a target for tumor differentiation therapy [78]. Similarly, USP1 is aberrantly overexpressed in various cancers and is closely associated with tumor development and progression, and is also generally upregulated in the BMCs of B-ALL patients. Both siRNA and pharmacological inhibitor (SJB3-019A) targeting USP1 can suppress ID1/AKT axis, inhibit B-ALL cell growth, and promote apoptosis [79]. In addition, other known USP1 inhibitors, such as pimozide, SJB2-043, and C527, can promote ID1 degradation and inhibit the growth of leukemic cell lines and primary AML patient-derived leukemic cells [80]. MLL-AF9 fusion protein overexpressed fetal liver (FL) cells and BMCs obtained from the *Id1*^*−/−*^ mice are used to perform serial replanting assays to mimic infant and postnatal MLL-AF9-driven leukemia, respectively. Interestingly, *Id1* deletion delays the development of fetal MLL-AF9-driven leukemia and increases the expression of MLL-AF9-driven leukemogenesis markers *HoxA9* and *Meis1* in HSPCs, but accelerates the leukemogenesis of postnatal leukemia with downregulated *HoxA9* and *Meis1*. *P21* depletion can rescue the effects of *Id1* deletion in both models, suggesting again that *p21* is a well-established target of *Id1*. This finding demonstrates the differential effects of a gene on fetal and postnatal MLL-AF9-mediated leukemogenesis for the first time; however, the molecular pathogenesis is largely unknown and needs to be further clarified [18, 81].

A recent study sheds light on how ID1 regulates leukemia progression in a noncell-autonomous manner since ID1 is widely expressed in various cells in the BM microenvironment, as described above. In the AML BM microenvironment, bone morphogenetic protein 6 (BMP6) secreted from LSCs induces high levels of ID1 in BMCs, especially in MSCs. ID1 directly interacts with Ring Finger Protein 4 (RNF4), an E3 ubiquitin ligase, which subsequently diminishes SP1 ubiquitination and leads to the release of Angiopoietin Like 7 (ANGPTL7) in MSCs to accelerate AML progression. Knocking out ID1 in MSCs can induce cell-cycle arrest, suppress proliferation of cocultured AML cells in vitro, and extend the lifespan of both AML-ETO- and MLL-AF9-driven AML mice in vivo [18].

### ID1 in other hematologic disorders

**Fig. 5:**
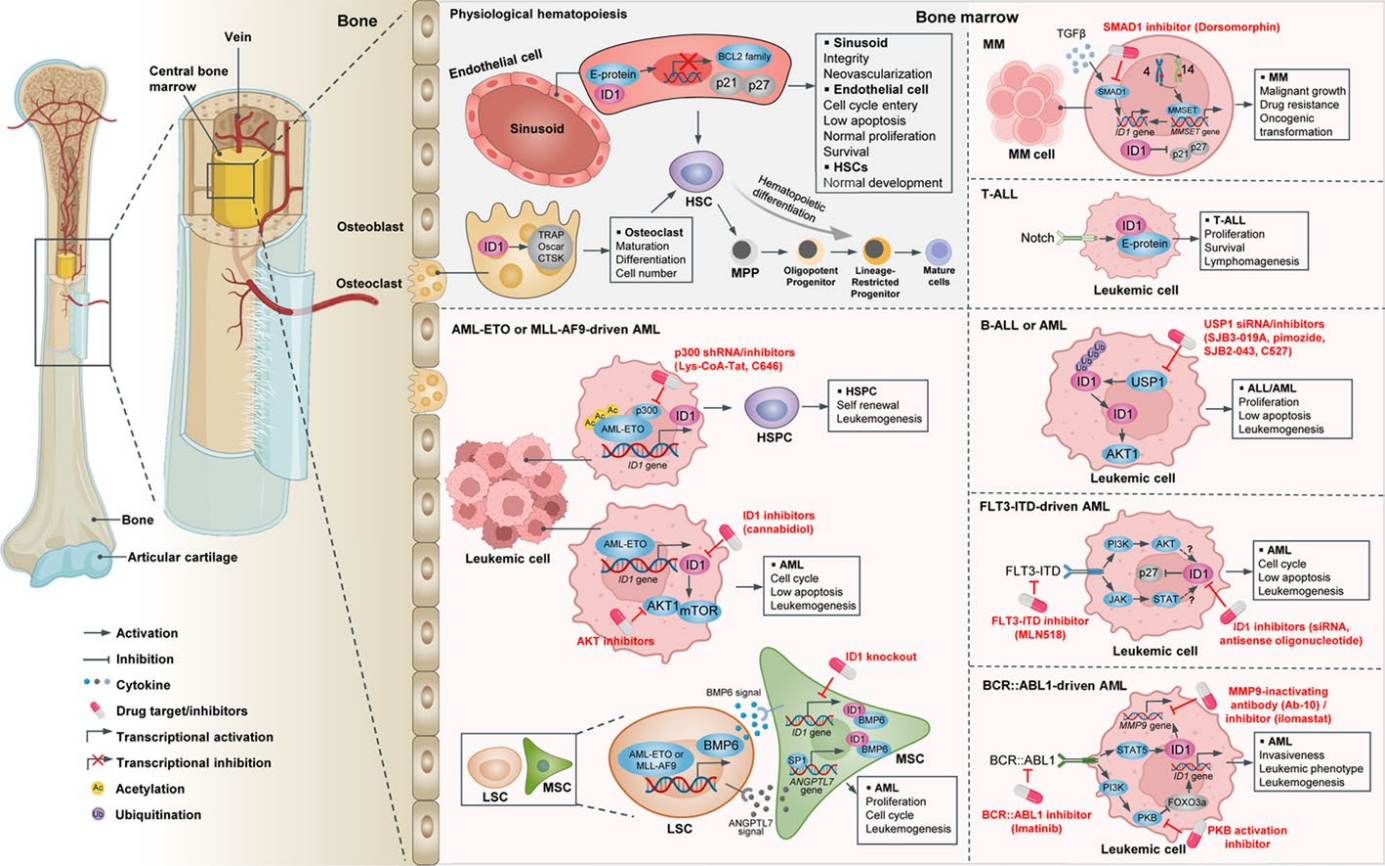
The mechanisms of ID1 involved in regulating physiological hematopoiesis and hematologic disorders in the bone marrow microenvironment. In the physiological bone marrow niche, proper expressions of ID1 in hematopoietic cells and stromal cells (including sinusoidal endothelial cells and osteoclasts) maintain hematopoietic homeostasis (in the grey background). In hematologic disorders, including multiple myeloma (MM), acute lymphoblastic leukemia (ALL), and acute myeloid leukemia (AML), aberrant ID1 overexpression induced by multiple pathogenic actors, including fusion proteins, cytokines, oncogenic kinases, ubiquitination, and oncogenic signaling pathways, can promote malignant transformation by regulating cell cycle, apoptosis, and invasion (in the pink background). HSPC: hematopoietic stem and progenitor cell; LSC: leukemia stem cell; MSC: mesenchymal stem cell; T-ALL: T cell ALL; B-ALL: B cell ALL

Both T cell-specific *Id1* expression and *Tcf3* ablation lead to T cell lymphoma formation in transgenic mice at high frequencies, while *Id1* gene disruption can partially rescue the survival of *Tcf3*-null mice [21, 50, 82, 83]. As a potent oncogenic factor, Notch pathway activation is beneficial but not indispensable for cooperating with *Id1* to promote T cell lymphomagenesis by enhancing the survival and proliferation of leukemic cells that express *Id1* [21]. One of the most frequent translocations in multiple myeloma, t(4;14), results in the overexpression of the oncogene multiple myeloma SET domain (MMSET). *ID1* expression is detected in 40% (6/15) of the t(4;14)^+^ MM patients and is significantly associated with t(4;14) translocation. *ID1* has been identified as a downstream transcriptional activation target of MMSET, facilitating oncogenic transformation in MM [84]. The high level of TGF-β secreted in the BM microenvironment of patients with MM activates the SMAD1-ID1-p21/p27 pathway to confer malignant growth properties and drug resistance upon MM cells [85]. The JAK2-STAT5 signaling cascade activated by the acquired mutation *JAK2*^V617F^ plays a pivotal role in the pathogenesis of multiple MPNs, such as polycythemia vera, essential thrombocythemia, and primary myelofibrosis. In murine fetal liver cells and patients’ erythroid cells of essential thrombocythemia patients, ID1 expression is positively correlated with *JAK2*^V617F^ mutation and is transcriptionally regulated by JAK2-STAT5 pathway to promote the erythroblast expansion and survival, and hence involved in the pathogenesis of erythrocytosis (Fig. 5) [20]. Therefore, ID1 expression may exert a broad malignant transformation role in other hematologic disorders besides leukemia.

## Discussion and perspectives

These well-established models for studying ID1’s role in the hematopoietic system have led to several key conclusions. First, in ESCs and iPSCs, ID1 exhibits dynamic expression, increasing during hemogenic specification but declining upon lineage commitment. ID1 acts as a direct negative regulator of hemogenic precursor transition to the hematopoietic lineage in ESCs and iPSCs, and helps maintain self-renewal and inhibit differentiation of ESCs (Fig. 2). Second, in HSCs and their immediate progeny, ID1 expression peaks in the most primitive LSK cells and declines along with the progression of differentiation and increasing maturity in hematopoietic cells, but is turned back on in GMPs and subsequent myeloid lineage cells (Fig. 2). Third, ID1 is widely expressed in hematopoietic and stromal cells in the BM niche, and regulates hematopoietic homeostasis. ID1 is pivotal for tri-lineage development and orchestrating progenitor cell fate between myeloid and lymphoid lineages. ID1 overexpression in HSPCs drives myeloid development at the expense of erythroid and lymphoid formation and even immortalizes myeloid progenitors. *ID1*-deficient BM microenvironment fails to support proper hematopoietic development (Fig. 5). In addition, *ID1* functions as an oncogene in the context of leukemia. Its aberrant overexpression correlates with poor clinical outcomes by driving malignant proliferation, invasion, and therapy resistance in leukemic cells (Fig. 5). Given these multi-roles, ID1 emerges as a promising therapeutic target for differentiation therapy in hematologic disorders.

Small-molecule inhibitors represent promising anticancer agents in development, offering broader clinical applicability than gene editing and serving as an alternative strategy for knocking down ID1 protein. Drugs targeting ID1 proteins must be selective for the ID1-bHLH interaction and do not affect bHLH dimerization. However, targeting nuclear protein–protein interactions, such as those between ID1 and HLH transcription factors, remains a formidable challenge for small-molecule drug development. The systemic delivery of an antisense molecule to ID1, peptide conjugate antisense oligonucleotide (ID1-PCAO), can effectively reduce ID1 protein levels in tumor endothelium to inhibit primary tumor growth and metastatic spread of lung cancer and breast cancer. However, ID1-PCAO has not yet been adapted to penetrate myeloid cells and has failed to be used for AML therapy [76, 86]. ID1 inhibitor CBD, a cannabinoid with a low toxicity profile, can downregulate ID1 expression and exhibit effective antileukemic outcomes. Unfortunately, CBD is a controlled substance and difficult to obtain, which limits its application. Given that ID1 promotes AKT1 phosphorylation through its C-terminal region without disrupting its interaction with bHLH, combined targeting of ID1 and AKT1 might be a promising therapeutic strategy for leukemia [76]. Recently, various novel inhibitors of USP1 with satisfactory anti-tumor benefits have been developed to induce ID1 degradation, cellular differentiation, and apoptosis in leukemic cells. Notably, a specific USP1 inhibitor, Pimozide, a US FDA-approved antipsychotic drug, exhibits potent anticancer effects in preclinical models of AML, T cell leukemia, and diffuse large B cell lymphoma, with minimal toxicity [76, 87, 88]. A recent study reported that trametinib-induced ID1 reduction helps create a less immunosuppressive tumor microenvironment and promotes the synergistic effect of trametinib combined with PD-1/PD-L1 blockade in lung adenocarcinoma, suggesting trametinib as a therapeutic alternative to target ID1 directly [89]. Similarly, ID1 expression participates in forming the BM immunosuppressive microenvironment in patients with ALL, indicating its potential as a target for novel immunotherapeutic combinations [67]. Despite these advances, translating ID1-targeted therapies into clinical practice will require substantial further research.

## Data Availability

Not applicable.

## Abbreviations:

ALL: Acute lymphoblastic leukemia
AML: Acute myeloid leukemia
APL: Acute promyelocytic leukemia
bp: Base pairs
bHLH: Basic helix-loop-helix
BMC: Bone marrow cell
BMT: Bone marrow transplantation
CBD: Cannabidiol
CEBPA: CCAAT/enhancer-binding protein-alpha
CFU: Colony forming unit
CLP: Common lymphoid progenitor
CMP: Common myeloid progenitor
DFS: Disease-free survival
DMSO: Dimethyl sulfoxide
EPO: Erythropoietin
ESC: Embryonic stem cell
FLT3L: FMS-related tyrosine kinase 3 ligand
GFP: Green fluorescent protein
GM-CSF: Granulocyte–macrophage colony-stimulating factor
GMP: Granulocyte-monocyte progenitor
HGF: Hematopoietic growth factor
HSC: Hematopoietic stem cell
HSPC: Hematopoietic stem and progenitor cell
ID1: Inhibitor of DNA binding 1
IFN-γ: Interferon-γ
IL-3: Interleukin 3
iPSC: Induced pluripotent stem cell
LMPP: Lymphoid-primed MPP
LSC: Leukemic stem cells
LT-HSC: Long-term HSC
MDS: Myelodysplastic syndrome
MEP: Megakaryocyte erythroid progenitor cell
MM: Multiple myeloma
MPN: Myeloproliferative neoplasm
MPP: Multipotent progenitor
MSC: Mesenchymal stem cell
OS: Overall survival
PB: Peripheral blood
qPCR: Quantitative polymerase chain reaction
SCF: Stem cell factor
shRNA: Short hairpin RNA
siRNA: Small interfering RNA
ST-HSC: Short-term HSC
TCF3: Transcription factor 3
TCR: T cell receptor
TNF-α: Tumor necrosis factor-α
TPO: Thrombopoietin
Treg: T regulatory cell

## References

[1] Massari ME, Murre C. Helix-loop-helix proteins: regulators of transcription in eucaryotic organisms. Mol Cell Biol. 2000;20(2):429–40. 10611221 10.1128/mcb.20.2.429-440.2000 PMC85097

[2] Sikder HA, Devlin MK, Dunlap S, Ryu B, Alani RM. Id proteins in cell growth and tumorigenesis. Cancer Cell. 2003;3(6):525–30. 12842081 10.1016/s1535-6108(03)00141-7

[3] Benezra R, Davis RL, Lockshon D, Turner DL, Weintraub H. The protein Id: a negative regulator of Helix-Loop-Helix DNA binding proteins. Cell. 1990;61(1):49–59. 2156629 10.1016/0092-8674(90)90214-y

[4] Roschger C, Cabrele C. The Id-protein family in developmental and cancer-associated pathways. Cell Commun Signal. 2017;15(1):7. 28122577 10.1186/s12964-016-0161-y PMC5267474

[5] Nehlin JO, Hara E, Kuo WL, Collins C, Campisi J. Genomic organization, sequence, and chromosomal localization of the human helix-loop-helix Id1 gene. Biochem Biophys Res Commun. 1997;231(3):628–34. 9070860 10.1006/bbrc.1997.6152

[6] Mathew S, Chen W, Murty VV, Benezra R, Chaganti RS. Chromosomal assignment of human ID1 and ID2 genes. Genomics. 1995;30(2):385–7. 8586447 10.1006/geno.1995.0037

[7] Deed RW, Hirose T, Mitchell EL, Santibanez-Koref MF, Norton JD. Structural organisation and chromosomal mapping of the human Id-3 gene. Gene. 1994;151(1–2):309–14. 7828896 10.1016/0378-1119(94)90676-9

[8] Pagliuca A, Bartoli PC, Saccone S, Della VG, Lania L. Molecular cloning of ID4, a novel dominant negative helix-loop-helix human gene on chromosome 6p21.3-p22. Genomics. 1995;27(1):200–3. 7665172 10.1006/geno.1995.1026

[9] Suh HC, Leeanansaksiri W, Ji M, Klarmann KD, Renn K, Gooya J, et al. Id1 immortalizes hematopoietic progenitors in vitro and promotes a myeloproliferative disease in vivo. Oncogene. 2008;27(42):5612–23. 18542061 10.1038/onc.2008.175 PMC3073486

[10] Chu YH, Lin JD, Nath S, Schachtrup C. Id proteins: emerging roles in CNS disease and targets for modifying neural stemcell behavior. Cell Tissue Res. 2022;387(3):433–49. 34302526 10.1007/s00441-021-03490-z PMC8975794

[11] Guo D, Yao B, Shao WW, Zuo JC, Chang ZH, Shi JX, et al. The critical role of YAP/BMP/ID1 axis on simulated microgravity-induced neural tube defects in human brain organoids. Adv Sci (Weinh). 2024;12(5):e2410188. 39656892 10.1002/advs.202410188 PMC11792043

[12] Shen H, Gao Y, Ge D, Tan M, Yin Q, Wei TW, et al. BRCC3 regulation of ALK2 in vascular smooth muscle cells: implication in pulmonary hypertension. Circulation. 2024;150(2):132–50. 38557054 10.1161/CIRCULATIONAHA.123.066430 PMC11230848

[13] Liu W, Gao L, Hou X, Feng S, Yan H, Pan H, et al. TWEAK signaling-induced ID1 expression drives malignant transformation of hepatic progenitor cells during hepatocarcinogenesis. Adv Sci. 2023;10(18):e2300350. 10.1002/advs.202300350 PMC10288241 37085918

[14] Huang YH, Hu J, Chen F, Lecomte N, Basnet H, David CJ, et al. Id1 mediates escape from TGFβ tumor suppression in pancreatic cancer. Cancer Discov. 2020;10(1):142–57. 31582374 10.1158/2159-8290.CD-19-0529 PMC6954299

[15] Phadte P, Bishnu A, Dey P, M M, Mehrotra M, Singh P, et al. Autophagy-mediated ID1 turnover dictates chemo-resistant fate in ovarian cancer stem cells. J Exp Clin Cancer Res. 2024;43(1):222. 39123206 10.1186/s13046-024-03147-z PMC11316295

[16] Pinho S, Frenette PS. Haematopoietic stem cell activity and interactions with the niche. Nat Rev Mol Cell Biol. 2019;20(5):303–20. 30745579 10.1038/s41580-019-0103-9 PMC6483843

[17] Suh HC, Ji M, Gooya J, Lee M, Klarmann KD, Keller JR. Cell-nonautonomous function of Id1 in the hematopoietic progenitor cell niche. Blood. 2009;114(6):1186–95. 19478045 10.1182/blood-2008-09-179788 PMC2723014

[18] Fei MY, Wang Y, Chang BH, Xue K, Dong F, Huang D, et al. The non-cell-autonomous function of ID1 promotes AML progression via ANGPTL7 from the microenvironment. Blood. 2023;142(10):903–17. 37319434 10.1182/blood.2022019537 PMC10644073

[19] Singh S, Sarkar T, Jakubison B, Gadomski S, Spradlin A, Gudmundsson KO, et al. Inhibitor of DNA binding proteins revealed as orchestrators of steady state, stress and malignant hematopoiesis. Front Immunol. 2022;13:934624. 35990659 10.3389/fimmu.2022.934624 PMC9389078

[20] Wood AD, Chen E, Donaldson IJ, Hattangadi S, Burke KA, Dawson MA, et al. ID1 promotes expansion and survival of primary erythroid cells and is a target of JAK2V617F-STAT5 signaling. Blood. 2009;114(9):1820–30. 19571317 10.1182/blood-2009-02-206573 PMC3942052

[21] Wang H, Peng V, Zhao Y, Sun X, Swarbrick A. Enhanced notch activation is advantageous but not essential for T cell lymphomagenesis in Id1 transgenic mice. PLoS ONE. 2012;7(2):e32944. 22393458 10.1371/journal.pone.0032944 PMC3290631

[22] Tam WF, Gu TL, Chen J, Lee BH, Bullinger L, Frohling S, et al. Id1 is a common downstream target of oncogenic tyrosine kinases in leukemic cells. Blood. 2008;112(5):1981–92. 18559972 10.1182/blood-2007-07-103010 PMC2518899

[23] Hong SH, Lee JH, Lee JB, Ji J, Bhatia M. ID1 and ID3 represent conserved negative regulators of human embryonic and induced pluripotent stem cell hematopoiesis. J Cell Sci. 2011;124(Pt 9):1445–52. 21486943 10.1242/jcs.077511

[24] Romero-Lanman EE, Pavlovic S, Amlani B, Chin Y, Benezra R. Id1 maintains embryonic stem cell self-renewal by up-regulation of Nanog and repression of Brachyury expression. Stem Cells Dev. 2012;21(3):384–93. 22013995 10.1089/scd.2011.0428

[25] Zhang Y, Gao S, Xia J, Liu F. Hematopoietic hierarchy-an updated roadmap. Trends Cell Biol. 2018;28(12):976–86. 29935893 10.1016/j.tcb.2018.06.001

[26] Mendelson A, Frenette PS. Hematopoietic stem cell niche maintenance during homeostasis and regeneration. Nat Med. 2014;20(8):833–46. 25100529 10.1038/nm.3647 PMC4459580

[27] Cheng H, Zheng Z, Cheng T. New paradigms on hematopoietic stem cell differentiation. Protein Cell. 2020;11(1):34–44. 31201709 10.1007/s13238-019-0633-0 PMC6949320

[28] Nimmo RA, May GE, Enver T. Primed and ready: understanding lineage commitment through single cell analysis. Trends Cell Biol. 2015;25(8):459–67. 26004869 10.1016/j.tcb.2015.04.004

[29] Liggett LA, Sankaran VG. Unraveling hematopoiesis through the lens of genomics. Cell. 2020;182(6):1384–400. 32946781 10.1016/j.cell.2020.08.030 PMC7508400

[30] Chen L, Sun Q, Li G, Huang Q, Chen S, Fu Y, et al. Redefining hematopoietic progenitor cells and reforming the hierarchy of hematopoiesis. bioRxiv. 2024. 10.1101/2023.01.27.524347.39803508

[31] Leeanansaksiri W, Wang H, Gooya JM, Renn K, Abshari M, Tsai S, et al. IL-3 induces inhibitor of DNA-binding protein-1 in hemopoietic progenitor cells and promotes myeloid cell development. J Immunol. 2005;174(11):7014–21. 15905544 10.4049/jimmunol.174.11.7014

[32] Jankovic V, Ciarrocchi A, Boccuni P, DeBlasio T, Benezra R, Nimer SD. Id1 restrains myeloid commitment, maintaining the self-renewal capacity of hematopoietic stem cells. Proc Natl Acad Sci U S A. 2007;104(4):1260–5. 17227850 10.1073/pnas.0607894104 PMC1783103

[33] Perry SS, Zhao Y, Nie L, Cochrane SW, Huang Z, Sun X. Id1, but not Id3, directs long-term repopulating hematopoietic stem-cell maintenance. Blood. 2007;110(7):2351–60. 17622570 10.1182/blood-2007-01-069914 PMC1988946

[34] Cochrane SW, Zhao Y, Welner RS, Sun XH. Balance between Id and E proteins regulates myeloid-versus-lymphoid lineage decisions. Blood. 2009;113(5):1016–26. 18927439 10.1182/blood-2008-06-164996 PMC2635070

[35] Buitenhuis M, van Deutekom HW, Verhagen LP, Castor A, Jacobsen SE, Lammers JW, et al. Differential regulation of granulopoiesis by the basic helix-loop-helix transcriptional inhibitors Id1 and Id2. Blood. 2005;105(11):4272–81. 15701714 10.1182/blood-2004-12-4883

[36] Lister J, Forrester WC, Baron MH. Inhibition of an erythroid differentiation switch by the helix-loop-helix protein Id1. J Biol Chem. 1995;270(30):17939–46. 7629100 10.1074/jbc.270.30.17939

[37] Cooper CL, Brady G, Bilia F, Iscove NN, Quesenberry PJ. Expression of the Id family helix-loop-helix regulators during growth and development in the hematopoietic system. Blood. 1997;89(9):3155–65. 9129018

[38] Quesenberry PJ, Iscove NN, Cooper C, Brady G, Newburger PE, Stein GS, et al. Expression of basic helix-loop-helix transcription factors in explant hematopoietic progenitors. J Cell Biochem. 1996;61(3):478–88. 8761952 10.1002/(SICI)1097-4644(19960601)61:3%3C478::AID-JCB15%3E3.0.CO;2-F

[39] Cooper CL, Newburger PE. Differential expression of Id genes in multipotent myeloid progenitor cells: Id-1 is induced by early- and late-acting cytokines while Id-2 is selectively induced by cytokines that drive terminal granulocytic differentiation. J Cell Biochem. 1998;71(2):277–85. 9779825 10.1002/(sici)1097-4644(19981101)71:2<277::aid-jcb12>3.0.co;2-i

[40] Kreider BL, Benezra R, Rovera G, Kadesch T. Inhibition of myeloid differentiation by the helix-loop-helix protein Id. Science. 1992;255(5052):1700–2. 1372755 10.1126/science.1372755

[41] Martin CH, Woll PS, Ni Z, Zúñiga-Pflücker JC, Kaufman DS. Differences in lymphocyte developmental potential between human embryonic stem cell and umbilical cord blood-derived hematopoietic progenitor cells. Blood. 2008;112(7):2730–7. 18621931 10.1182/blood-2008-01-133801 PMC2556609

[42] Shoji W, Yamamoto T, Obinata M. The helix-loop-helix protein Id inhibits differentiation of murine erythroleukemia cells. J Biol Chem. 1994;269(7):5078–84. 8106486

[43] Fraszczak J, Helness A, Chen R, Vadnais C, Robert F, Khandanpour C, et al. Threshold levels of Gfi1 maintain E2A activity for B cell commitment via repression of Id1. PLoS ONE. 2016;11(7):e0160344. 27467586 10.1371/journal.pone.0160344 PMC4965025

[44] Spooner CJ, Cheng JX, Pujadas E, Laslo P, Singh H. A recurrent network involving the transcription factors PU.1 and Gfi1 orchestrates innate and adaptive immune cell fates. Immunity. 2009;31(4):576–86. 19818654 10.1016/j.immuni.2009.07.011 PMC4373467

[45] Cammenga J, Mulloy JC, Berguido FJ, MacGrogan D, Viale A, Nimer SD. Induction of C/EBPα activity alters gene expression and differentiation of human CD34+ cells. Blood. 2003;101(6):2206–14. 12406909 10.1182/blood-2002-05-1546

[46] Chan AS, Jensen KK, Skokos D, Doty S, Lederman HK, Kaplan RN, et al. Id1 represses osteoclast-dependent transcription and affects bone formation and hematopoiesis. PLoS ONE. 2009;4(11):e7955. 19956687 10.1371/journal.pone.0007955 PMC2776978

[47] Singh SK, Singh S, Gadomski S, Sun L, Pfannenstein A, Magidson V, et al. Id1 ablation protects hematopoietic stem cells from stress-induced exhaustion and aging. Cell Stem Cell. 2018;23(2):252–65. 30082068 10.1016/j.stem.2018.06.001 PMC6149219

[48] Cochrane SW, Zhao Y, Perry SS, Urbaniak T, Sun X. Id1 has a physiological role in regulating early B lymphopoiesis. Cell Mol Immunol. 2011;8(1):41–9. 21200383 10.1038/cmi.2010.58 PMC3058379

[49] Sun XH. Constitutive expression of the Id1 gene impairs mouse B cell development. Cell. 1994;79(5):893–900. 8001126 10.1016/0092-8674(94)90078-7

[50] Kim D, Peng XC, Sun XH. Massive apoptosis of thymocytes in T-cell-deficient Id1 transgenic mice. Mol Cell Biol. 1999;19(12):8240–53. 10567549 10.1128/mcb.19.12.8240 PMC84908

[51] Kim D, Xu M, Nie L, Peng XC, Jimi E, Voll RE, et al. Helix-loop-helix proteins regulate pre-TCR and TCR signaling through modulation of Rel/NF-kappaB activities. Immunity. 2002;16(1):9–21. 11825562 10.1016/s1074-7613(02)00264-9

[52] Yang Y, Liou H, Sun X. Id1 potentiates NF-kappaB activation upon T cell receptor signaling. J Biol Chem. 2006;281(46):34989–96. 17012234 10.1074/jbc.M608078200

[53] Liu C, Jin R, Wang H, Tang H, Liu Y, Qian X, et al. Id1 expression promotes peripheral CD4+ T cell proliferation and survival upon TCR activation without co-stimulation. Biochem Biophys Res Commun. 2013;436(1):47–52. 23707719 10.1016/j.bbrc.2013.05.054 PMC3955101

[54] Liu C, Wang HC, Yu S, Jin R, Tang H, Liu YF, et al. Id1 expression promotes T regulatory cell differentiation by facilitating TCR costimulation. J Immunol. 2014;193(2):663–72. 24920844 10.4049/jimmunol.1302554 PMC4082736

[55] Lee WH, Hong KJ, Li HB, Lee GR. Transcription factor Id1 plays an essential role in Th9 cell differentiation by inhibiting Tcf3 and Tcf4. Adv Sci (Weinh). 2023;10(35):e2305527. 37867222 10.1002/advs.202305527 PMC10724384

[56] Lyden D, Young AZ, Zagzag D, Yan W, Gerald W, O’Reilly R, et al. Id1 and Id3 are required for neurogenesis, angiogenesis and vascularization of tumour xenografts. Nature. 1999;401(6754):670–7. 10537105 10.1038/44334

[57] James D, Nam HS, Seandel M, Nolan D, Janovitz T, Tomishima M, et al. Expansion and maintenance of human embryonic stem cell-derived endothelial cells by TGFbeta inhibition is Id1 dependent. Nat Biotechnol. 2010;28(2):161–6. 20081865 10.1038/nbt.1605 PMC2931334

[58] Gadomski S, Singh SK, Singh S, Sarkar T, Klarmann KD, Berenschot M, et al. Id1 and Id3 maintain steady-state hematopoiesis by promoting sinusoidal endothelial cell survival and regeneration. Cell Rep. 2020;31(4):107572. 32348770 10.1016/j.celrep.2020.107572 PMC8459380

[59] Zhou JD, Yang L, Zhu XW, Wen XM, Yang J, Guo H, et al. Clinical significance of up-regulated ID1 expression in Chinese de novo acute myeloid leukemia. Int J Clin Exp Pathol. 2015;8(5):5336–44. 26191235 PMC4503106

[60] Tang R, Hirsch P, Fava F, Lapusan S, Marzac C, Teyssandier I, et al. High Id1 expression is associated with poor prognosis in 237 patients with acute myeloid leukemia. Blood. 2009;114(14):2993–3000. 19643984 10.1182/blood-2009-05-223115

[61] Zhao Q, Wang Y, Yu D, Leng JY, Zhao Y, Chu M, et al. Comprehensive analysis of ID genes reveals the clinical and prognostic value of ID3 expression in acute myeloid leukemia using bioinformatics identification and experimental validation. BMC Cancer. 2022;22(1):1229. 36443709 10.1186/s12885-022-10352-6 PMC9707109

[62] Damm F, Wagner K, Görlich K, Morgan M, Thol F, Yun H, et al. ID1 expression associates with other molecular markers and is not an independent prognostic factor in cytogenetically normal acute myeloid leukaemia. Br J Haematol. 2012;158(2):208–15. 22568493 10.1111/j.1365-2141.2012.09144.x

[63] Wagner K, Zhang P, Rosenbauer F, Drescher B, Kobayashi S, Radomska HS, et al. Absence of the transcription factor CCAAT enhancer binding protein alpha results in loss of myeloid identity in bcr/abl-induced malignancy. Proc Natl Acad Sci U S A. 2006;103(16):6338–43. 16606850 10.1073/pnas.0508143103 PMC1458879

[64] Kantzer CG, Yang W, Grommisch D, Patil KV, Mak KH, Shirokova V, et al. ID1 and CEBPA coordinate epidermal progenitor cell differentiation. Development. 2022;149(22):dev201262. 36330928 10.1242/dev.201262 PMC9845743

[65] Tochareontanaphol C, Sinthuwiwat T, Buathong B, Thita T, Promso S, Paca-Uccaralertkun S. New mutations of the ID1 gene in acute myeloid leukemia patients. Pathobiology. 2015;82(1):43–7. 25766257 10.1159/000370243

[66] Cruz-Rodriguez N, Combita AL, Enciso LJ, Quijano SM, Pinzon PL, Lozano OC, et al. High expression of ID family and IGJ genes signature as predictor of low induction treatment response and worst survival in adult Hispanic patients with B-acute lymphoblastic leukemia. J Exp Clin Cancer Res. 2016;35:64. 27044543 10.1186/s13046-016-0333-z PMC4820984

[67] Poveda-Garavito N, Orozco Castaño CA, Torres-Llanos Y, Cruz-Rodriguez N, Parra-Medina R, Quijano S, et al. ID1 and ID3 functions in the modulation of the tumour immune microenvironment in adult patients with B-cell acute lymphoblastic leukaemia. Front Immunol. 2024;15:1473909. 39676870 10.3389/fimmu.2024.1473909 PMC11638060

[68] Wen XM, Zhang TJ, Ma JC, Zhou JD, Xu ZJ, Zhu XW, et al. Establishment and molecular characterization of decitabine-resistant K562 cells. J Cell Mol Med. 2019;23(5):3317–24. 30793488 10.1111/jcmm.14221 PMC6484323

[69] Ma J, Wen X, Xu Z, Xia P, Jin Y, Lin J, et al. Abnormal regulation of miR-29b-ID1 signaling is involved in the process of decitabine resistance in leukemia cells. Cell Cycle. 2023;22(10):1215–31. 37032592 10.1080/15384101.2023.2200312 PMC10193880

[70] Yu WP, Scott SA, Dong WF. Induction of ID1 expression and apoptosis by the histone deacetylase inhibitor (trichostatin A) in human acute myeloid leukaemic cells. Cell Prolif. 2008;41(1):86–97. 18211287 10.1111/j.1365-2184.2007.00499.x PMC6496488

[71] Nigten J, Breems-de RM, Erpelinck-Verschueren CA, Nikoloski G, van der Reijden BA, van Wageningen S, et al. ID1 and ID2 are retinoic acid responsive genes and induce a G0/G1 accumulation in acute promyelocytic leukemia cells. Leukemia. 2005;19(5):799–805. 15744343 10.1038/sj.leu.2403699

[72] Bhanumathy KK, Balagopal A, Vizeacoumar FS, Vizeacoumar FJ, Freywald A, Giambra V. Protein tyrosine kinases: their roles and their targeting in leukemia. Cancers (Basel). 2021;13(2):184. 33430292 10.3390/cancers13020184 PMC7825731

[73] Nieborowska-Skorska M, Hoser G, Rink L, Malecki M, Kossev P, Wasik MA, et al. Id1 transcription inhibitor-matrix metalloproteinase 9 axis enhances invasiveness of the breakpoint cluster region/abelson tyrosine kinase-transformed leukemia cells. Cancer Res. 2006;66(8):4108–16. 16618731 10.1158/0008-5472.CAN-05-1584

[74] Birkenkamp KU, Essafi A, van der Vos KE, Da Costa M, Hui RCY, Holstege F, et al. Foxo3a induces differentiation of Bcr-Abl-transformed cells through transcriptional down-regulation of Id1. J Biol Chem. 2007;282(4):2211–20. 17132628 10.1074/jbc.M606669200

[75] Wang L, Gural A, Sun XJ, Zhao X, Perna F, Huang G, et al. The leukemogenicity of AML1-ETO is dependent on site-specific lysine acetylation. Science. 2011;333(6043):765–9. 21764752 10.1126/science.1201662 PMC3251012

[76] Wang L, Man N, Sun XJ, Tan Y, Garcia-Cao M, Liu F, et al. Regulation of AKT signaling by Id1 controls t(8;21) leukemia initiation and progression. Blood. 2015;126(5):640–50. 26084673 10.1182/blood-2015-03-635532 PMC4520879

[77] McAllister SD, Christian RT, Horowitz MP, Garcia A, Desprez P. Cannabidiol as a novel inhibitor of Id-1 gene expression in aggressive breast cancer cells. Mol Cancer Ther. 2007;6(11):2921–7. 18025276 10.1158/1535-7163.MCT-07-0371

[78] Williams SA, Maecker HL, French DM, Liu J, Gregg A, Silverstein LB, et al. USP1 deubiquitinates ID proteins to preserve a mesenchymal stem cell program in osteosarcoma. Cell. 2011;146(6):918–30. 21925315 10.1016/j.cell.2011.07.040

[79] Kuang X, Xiong J, Lu T, Wang W, Zhang Z, Wang J. Inhibition of USP1 induces apoptosis via ID1/AKT pathway in B-cell acute lymphoblastic leukemia cells. Int J Med Sci. 2021;18(1):245–55. 33390793 10.7150/ijms.47597 PMC7738972

[80] Mistry H, Hsieh G, Buhrlage SJ, Huang M, Park E, Cuny GD, et al. Small-molecule inhibitors of USP1 target ID1 degradation in leukemic cells. Mol Cancer Ther. 2013;12(12):2651–62. 24130053 10.1158/1535-7163.MCT-13-0103-T PMC4089878

[81] Man N, Sun XJ, Tan Y, Garcia-Cao M, Liu F, Cheng G, et al. Differential role of Id1 in MLL-AF9-driven leukemia based on cell of origin. Blood. 2016;127(19):2322–6. 26944543 10.1182/blood-2015-11-677708 PMC4865589

[82] Dudley DD, Wang HC, Sun XH. Hes1 potentiates T cell lymphomagenesis by up-regulating a subset of notch target genes. PLoS ONE. 2009;4(8):e6678. 19688092 10.1371/journal.pone.0006678 PMC2722736

[83] Yan W, Young AZ, Soares VC, Kelley R, Benezra R, Zhuang Y. High incidence of T-cell tumors in E2A-null mice and E2A/Id1 double-knockout mice. Mol Cell Biol. 1997;17(12):7317–27. 9372963 10.1128/mcb.17.12.7317 PMC232588

[84] Hudlebusch HR, Theilgaard Mönch K, Lodahl M, Johnsen HE, Rasmussen T. Identification of ID-1 as a potential target gene of MMSET in multiple myeloma. Br J Haematol. 2005;130(5):700–8. 16115125 10.1111/j.1365-2141.2005.05664.x

[85] Wu J, Zhang M, Faruq O, Zacksenhaus E, Chen W, Liu A, et al. Smad1 as a biomarker and potential therapeutic target in drug-resistant multiple myeloma. Biomark Res. 2021;9(1):48. 34134766 10.1186/s40364-021-00296-7 PMC8207655

[86] Henke E, Perk J, Vider J, de Candia P, Chin Y, Solit DB, et al. Peptide-conjugated antisense oligonucleotides for targeted inhibition of a transcriptional regulator in vivo. Nat Biotechnol. 2008;26(1):91–100. 18176556 10.1038/nbt1366

[87] Li XY, Wu JC, Liu P, Li ZJ, Wang Y, Chen BY, et al. Inhibition of USP1 reverses the chemotherapy resistance through destabilization of MAX in the relapsed/refractory B-cell lymphoma. Leukemia. 2023;37(1):164–77. 36352191 10.1038/s41375-022-01747-2 PMC9883169

[88] Ishikawa C, Mori N. The antipsychotic drug pimozide is effective against human T-cell leukemia virus type 1-infected T cells. Eur J Pharmacol. 2021;908:174373. 34303663 10.1016/j.ejphar.2021.174373

[89] Puyalto A, Rodriguez-Remirez M, Lopez I, Macaya I, Guruceaga E, Olmedo M, et al. Trametinib sensitizes *KRAS*-mutant lung adenocarcinoma tumors to PD-1/PD-L1 axis blockade via Id1 downregulation. Mol Cancer. 2024;23(1):78. 38643157 10.1186/s12943-024-01991-3 PMC11031964

